# mRNA/microRNA gene expression profile in microsatellite unstable colorectal cancer

**DOI:** 10.1186/1476-4598-6-54

**Published:** 2007-08-23

**Authors:** Giovanni Lanza, Manuela Ferracin, Roberta Gafà, Angelo Veronese, Riccardo Spizzo, Flavia Pichiorri, Chang-gong Liu, George A Calin, Carlo M Croce, Massimo Negrini

**Affiliations:** 1 Department of Experimental and Diagnostic Medicine and Interdepartment Center for Cancer Research, University of Ferrara, Ferrara, Italy; 2 Department of Molecular Virology, Immunology and Medical Genetics and Comprehensive Cancer Center, Ohio State University, Columbus, OH, USA

## Abstract

**Background:**

Colorectal cancer develops through two main genetic instability pathways characterized by distinct pathologic features and clinical outcome.

**Results:**

We investigated colon cancer samples (23 characterized by microsatellite stability, MSS, and 16 by high microsatellite instability, MSI-H) for genome-wide expression of microRNA (miRNA) and mRNA. Based on combined miRNA and mRNA gene expression, a molecular signature consisting of twenty seven differentially expressed genes, inclusive of 8 miRNAs, could correctly distinguish MSI-H versus MSS colon cancer samples. Among the differentially expressed miRNAs, various members of the oncogenic miR-17-92 family were significantly up-regulated in MSS cancers. The majority of protein coding genes were also up-regulated in MSS cancers. Their functional classification revealed that they were most frequently associated with cell cycle, DNA replication, recombination, repair, gastrointestinal disease and immune response.

**Conclusion:**

This is the first report that indicates the existence of differences in miRNA expression between MSS versus MSI-H colorectal cancers. In addition, the work suggests that the combination of mRNA/miRNA expression signatures may represent a general approach for improving bio-molecular classification of human cancer.

## Background

Colorectal cancer develops through two main genetic pathways characterized by different forms of genomic instability [1]. Most tumors are generated by the chromosomal instability (CIN) pathway and display marked cytogenetic abnormalities, aneuploidy and allelic losses at multiple chromosomal arms. CIN is probably caused by various molecular mechanisms, but the underlying genetic alterations are still poorly defined. About 15% of colorectal carcinomas develop through the microsatellite instability (MSI) pathway. MSI tumors show stable karyotype, low frequencies of allelic losses and diploid nuclear DNA content. MSI results from defects in the DNA mismatch repair system (MMR) [2]. In HNPCC, MSI is produced by germline mutations of one of the MMR genes (MLH1, MSH2 and less frequently MSH6 and PMS2) with somatic inactivation of the wild-type allele [3,4]. In sporadic tumors, MMR deficiency is near always determined by epigenetic inactivation of the MLH1 gene by biallelic promoter methylation [5-7]. MSI colorectal adenocarcinomas display distinctive pathologic features, such as proximal location, poor differentiation, frequent mucinous and medullary phenotype, and marked peritumoral and intratumoral lymphocytic infiltration [8-10]. MSI carcinomas have a more favorable clinical outcome than non-MSI tumors and the survival advantage conferred by the MSI phenotype is independent of tumor stage and other clinical and pathological variables [11-13]. In addition, MMR-deficient cancer cells are thought to be less responsive to 5-fluorouracil and other chemotherapeutic agents in vitro and in vivo [14-16].

Gene expression analysis based on genome-wide microarrays has been largely used to characterize human cancers. This approach allowed the identification of genes important in tumorigenesis. Furthermore, the discovery of gene expression signatures characteristic of distinctive clinico-pathological features suggested that expression profiles could be used for molecular classification of human cancer [17-20]. Microarray tools have been recently enriched by the development of platforms for the analysis of microRNAs (miRNA) expression [21,22]. miRNAs are a class of small non-coding RNAs involved in temporal and tissue-specific eukaryotic gene regulation [23]. Comparison between human cancers and their normal counterparts revealed that miRNAs exhibit differential expression profiles in normal versus cancer tissues [24-29]. These studies revealed that some human miRNAs are consistently deregulated in human cancer, suggesting a role in tumorigenesis either as oncogenes or tumor suppressor genes [30-32]. Unique miRNA expression signatures were found to be associated with bio-molecular and prognostic characteristics of human lung cancer and chronic lymphocytic leukemia [24,33], indicating that miRNA signatures could be used to define biological or clinical features of human cancers. Known function of mammalian miRNAs is to post-transcriptionally regulate target mRNAs, implying that the combination of miRNAs and mRNAs expression may better represent the transcriptional program that dictates normal and tumor cell characteristics. Here, we identified differentially expressed miRNAs and mRNAs able to distinguish colon cancers with or without microsatellite instability.

## Results

We analyzed miRNA and mRNA expression using microarrays in a set of microsatellite stable (MSS) and unstable (MSI-H) colorectal cancers (23 MSS and 16 MSI-H) (Table 1) with the aim of recognizing the most significant differences in gene expression. In particular, this is the first study that investigates the differences in miRNAs expression between MSS and MSI tumors.

**Table 1:** Clinical and bio-pathological features of colorectal carcinomas employed in the study.

**Feature**	**No.**	**MSS**	**(%)**	**MSI-H**	**(%)**	P-value*
Sex						
Male	21	15	(65.2)	6	(37.5)	NS
Female	18	8	(34.8)	10	(62.5)	
						
Age (years)						
<55	3	2	(8.7)	1	(6.3)	NS
55–70	11	9	(39.1)	2	(12.5)	
>70	25	12	(52.2)	13	(81.3)	
						
Tumor site						
Proximal colon	18	5	(21.7)	13	(81.3)	<0.001
Distal colon	21	18	(78.3)	3	(18.7)	
						
Tumor stage (TNM)						
I	2	1	(4.3)	1	(6.3)	NS
II	16	9	(39.2)	7	(43.8)	
III	13	7	(30.4)	6	(37.5)	
IV	8	6	(26.1)	2	(12.5)	
						
Tumor type						
Adenocarcinoma	29	22	(95.7)	7	(43.7)	<0.001
Mucinous adenocarcinoma	10	1	(4.3)	9	(56.3)	
						
Grade of differentiation						
Well/moderate	29	22	(95.7)	7	(43.7)	<0.001
Poor	10	1	(4.3)	9	(56.3)	
						
MMR protein expression						
MLH1 negative	15	0		15	(93.8)	<0,001
MSH2 negative	1	0		1	(6.2)	
MLH1/MSH2 positive	23	23	(100)	0		

* Evaluated with Chi-square test or the Fisher's Exact test

NS, not significant

We carried out initial studies on miRNA and mRNA-chip data separately. The analysis of miRNA expression profiles of MSI-H versus MSS tumors identified 14 differentially expressed miRNAs (p < 0.05). Additional file 1 provides the list of deregulated miRNAs and their mean expression values and standard errors in the two groups. The analysis of mRNA expression profiles of MSI-H versus MSS identified a large number of differentially expressed genes (451 genes at p < 0.05; Additional file 2). By using the more stringent Bonferroni multiple testing correction at p < 0.05, a sub-list of 72 differentially expressed mRNAs was identified (Additional file 3).

**Figure 1:**
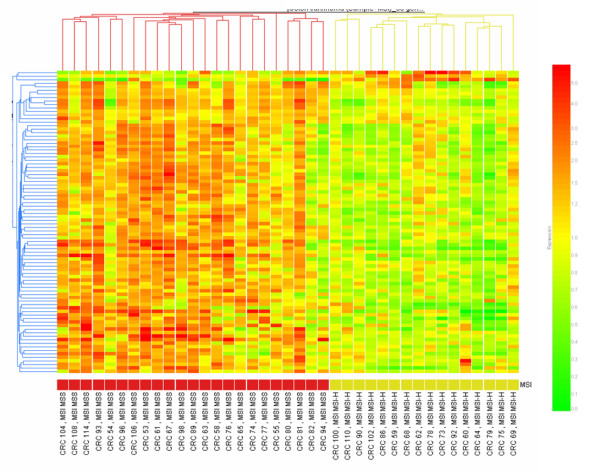
Supervised hierarchical clustering of 39 colon cancer samples (23 MSS and 16 MSI-H) using expression data from the list of 86 differentially expressed mRNA/miRNA genes described in Supplemental Tables 1 and 3. Columns display the clustering of tumor samples; rows the clustering of genes. MSI status is indicated by colored squares: red for MSS and yellow for MSI-H tumor samples. All sample were properly assigned to the correct class. The expression intensity of each gene in each sample varies from red to green: red color means an expression value over the average across samples, green color the opposite. For quantification of color codes, see color-bar on the right.

To visually assess the ability of the differentially expressed miRNAs and mRNAs to distinguish MSS versus MSI-H tumors, we applied the combination of the 86 differentially expressed miRNAs and mRNAs to perform a supervised cluster analysis. Using this combined set of genes, a perfect classification of the tumor samples resulted (Figure 1). The combination of the two sets of genes resulted also in an improvement in the separation of the MSS versus the MSI tumor samples in comparison to the individual sets (see Additional files 4 and 5).

**Figure 2:**
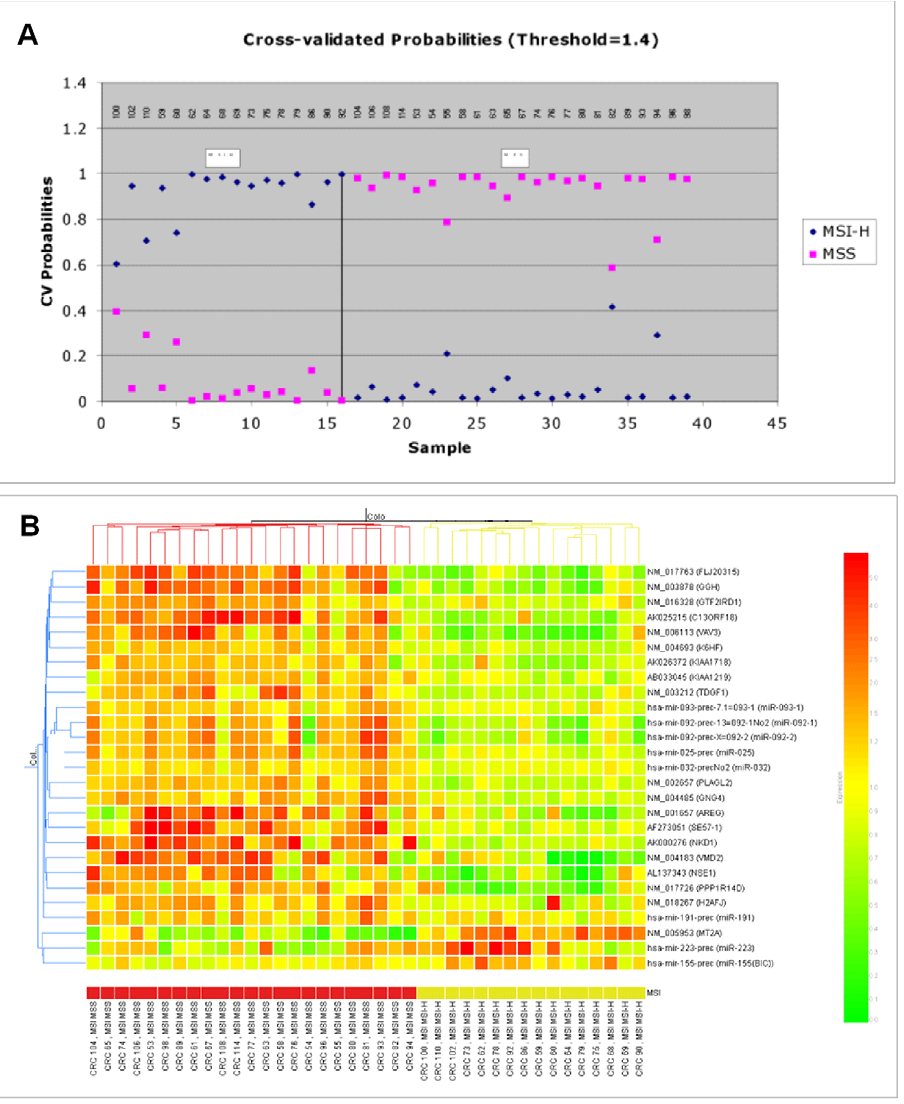
Prediction of microsatellite instability. (**A**) Prediction of MSI status for 39 samples using the PAM cross-validation procedure. The graphical representation shows the probabilities (0.0 to 1.0) of each tumor for being MSS (pink dot) or MSI-H (blue dot). All tumors were correctly assigned. (**B**) Supervised hierarchical clustering of colon cancer samples using expression data from the 27 predictor mRNA/miRNA genes described in Table 2. Using the predictors for the cluster analysis, a good separation among colorectal cancers in MSS (red) and MSI-H (yellow) groups was achieved. Expression intensity is shown as described in Figure 1.

A smaller selected signature of best predictors of microsatellite status was generated by applying Support Vector Machine (SVM) (GeneSpring software) and Prediction Analysis of Microarrays (PAM) algorithms to the 86 differentially expressed miRNAs and mRNAs: 27 genes (inclusive of mRNAs and miRNAs) were identified as predictors by at least one of the two algorithms (Table 2). Cluster analysis with these 27 miRNA/mRNAs still produced a perfect separation between the two tumor classes, and a PAM plot displayed a correct predictions for all the samples (Figure 2). The prediction of MSI status obtained with 72 mRNAs or 14 miRNAs signatures did not perform equally well when tested independently. Of the 39 samples, the miRNA-only signature could correctly predict 36 samples using SVM or 33 using PAM algorithms, and the mRNA-only signature could correctly predict 38 samples using SVM or 39 using PAM.

**Table 2:** Predictors of microsatellite status identified by Support Vector Machine and PAM algorithms.

**Probe**	**Symbol**		**Average expression**		**p-value**	SVM* strength	PAM* score	
		**Gene name**	**MSS**	**MSI-H**			**MSI-H**	**MSS**
hsa-mir-223-prec	miR-223		0.97	1.84	4.69E-02	-	0.4929	-0.3429
hsa-mir-155-prec	miR-155 (BIC)		0.97	1.29	2.34E-02	-	0.4439	-0.3088
AL137343	FAM84A	family with sequence similarity 84, member A	1.51	0.45	1.07E-02	-	-0.0208	0.0145
NM_004183	VMD2	vitelliform macular dystrophy 2	2.09	0.38	8.73E-03	-	-0.1832	0.1274
NM_006113	VAV3	vav 3 oncogene	1.62	0.47	5.98E-03	13.96	-0.0533	0.0371
NM_005953	MT2A	metallothionein 2A	0.57	1.92	5.93E-03	16.34	0.8995	-0.6258
AK026372	KIAA1718		1.27	0.72	5.09E-03	16.02	-	-
hsa-mir-191-prec	miR-191		1.28	1.00	4.74E-03	-	0.0377	-0.0263
NM_017726	PPP1R14D	protein phosphatase 1, regulatory (inhibitor) subunit 14D	1.31	0.52	3.88E-03	14.58	-	-
NM_003212	TDGF1	teratocarcinoma-derived growth factor 1	1.49	0.76	3.37E-03	16.49	-	-
AF273051	CCDC68	coiled-coil domain containing 68	2.21	0.84	2.49E-03	15.87	-0.0541	0.0376
NM_016328	GTF2IRD1	GTF2I repeat domain containing 1	1.49	0.87	2.13E-03	13.13	-	-
NM_001657	AREG	amphiregulin	2.10	0.69	1.04E-03	-	-0.0725	0.0504
AB033045	KIAA1219	hypothetical protein LOC57148	1.33	0.83	8.11E-04	15.87	-	-
hsa-mir-032-precNo2	miR-032		1.17	0.91	6.48E-04	14.02	0.0732	-0.0509
NM_004485	GNG4	guanine nucleotide binding protein (G protein), gamma 4	1.46	0.81	6.47E-04	16.49	-	-
NM_018267	H2AFJ	H2A histone family, member J	1.43	0.92	6.47E-04	13.96	-	-
AK000276	NKD1	naked cuticle homolog 1	2.47	0.77	4.55E-04	17.48	-0.1542	0.1072
NM_003878	GGH	gamma-glutamyl hydrolase	2.10	0.61	7.97E-05	15.87	-0.2232	0.1553
AK025215	C13ORF18	chromosome 13 open reading frame 18	2.41	0.66	4.40E-05	16.49	-0.3093	0.2152
NM_017763	RNF43	ring finger protein 43	2.10	0.57	2.91E-05	17.48	-0.2886	0.2008
NM_004693	K6HF	cytokeratin type II	1.24	0.80	2.91E-05	14.6	-	-
NM_002657	PLAGL2	pleiomorphic adenoma gene-like 2	1.37	0.76	2.91E-05	19.32	-	-
hsa-mir-025-prec	miR-025		1.52	0.84	2.14E-05	17.48	-	-
hsa-mir-092-prec-13=092-1No2	miR-092-1		1.50	0.65	1.87E-05	17.48	-	-
hsa-mir-092-prec-X=092-2	miR-092-2		1.60	0.64	1.87E-05	14.43	-	-
hsa-mir-093-prec-7.1=093-1	miR-093-1		1.23	0.9	1.87E-05	-	0.0084	-0.0058

* SVM: Support Vector Machine; PAM: Prediction Analysis of Microarray

**Figure 3:**
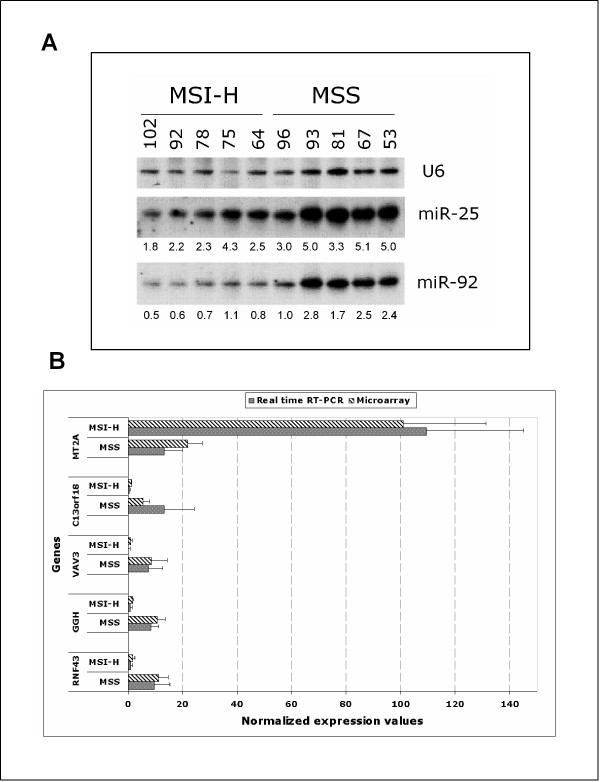
Confirmation of microarray data. (**A**) Northern blot analysis of miRNAs miR-25 and miR-92. As expected from microarray data, Northern blot analysis confirmed that these miRNAs were up-regulated in MSS samples. miRNA/U6 ratio of densitometric intensities is shown below each miRNA lane. (**B**) Quantitative Real time RT-PCR validation of microarray results. Microarray expression values of a selected panel of 5 genes differentially expressed between MSS and MSI-H groups of colorectal cancers were validated with quantitative real-time PCR. Values for microarray data were normalized on chip 50th percentile while for real-time PCR values are equal to ΔCt*10^6.

Confirmation of the differential expression for some of the signature genes was achieved by Northern blot to validate miRNA expression and Real time RT-PCR to validate expression of mRNAs. Figure 3A shows the expression of miR-25 and miR-92 as detected by Northern blot. This analysis confirmed that the expression of these miRNAs is increased in MSS samples as detected by microarray analysis. Similarly, the evaluation of expression of 5 protein-coding genes showed an overlap with the expression values obtained by microarray analysis (Figure 3B).

## Discussion

We report the analysis of the combined miRNA/mRNA expression for the discrimination of MSI-H versus MSS human colon cancer. Because colorectal tumors characterized by MSI are distinct from MSS tumors in many molecular aspects, such the association with the methylator phenotype, which is responsible for MLH1 methylation, the higher frequency of BRAF mutations and the lower frequencies of KRAS, APC and TP53 mutations, MSI and MSS colon cancers represent tumors with a different molecular background. Thus, it is reasonable that their overall gene expression pattern (including both mRNAs and microRNAs) might be affected by any of the above mentioned mechanisms. Indeed, our analyses identified 14 miRNAs and 451 mRNAs differentially expressed between the two genetically distinct colon cancer classes. These results not only indicate the existence of a mRNA/miRNA gene expression profile able to distinguish MSS versus MSI colon cancers, but also indicate that the combination of miRNA/mRNA gene expression data can distinguish microsatellite instability status of human colorectal cancers better than each RNA class independently, suggesting that the combination of mRNA and miRNA expression may potentially represent a general approach for improving characterization and classification of bio-molecular and possibly clinical traits associated with human cancer.

We also examined the prediction power of published lists of genes differentially expressed between MSI-H and MSS colorectal cancers [34-36] against our data set. Two published lists describe differentially expressed genes (100 genes in the report from Di Pietro et al. 2005 [36] and 542 genes from Banerjea et al. 2004 [35]) and one is a short list of predictors (9 genes in Kruhoffer et al. 2005 [34]). Although these studies were all performed on Affymetrix platforms, they generated only partially overlapping results. The list of genes from Di Pietro et al. [36] was the one that better correlated with our results. Twenty two of their 100 genes were present in our list of differentially expressed genes and, for all the remaining genes, expression tendency completely matched our data. A cluster analysis of our data set using the 100 genes from the Di Pietro's paper could generate a good, although not perfect, separation between MSS versus MSI-H tumors and performed well in the prediction of our samples (36 of 39 samples were correctly classified using the SVM prediction) (data not shown). These results indicate that a similar set of genes emerged as differentially expressed between MSS and MSI-H colon cancers in the Di Pietro's and our studies, with discrepancies possibly due to small statistical differences. Discrepancies between results obtained with different microarray platforms are not uncommon and are mainly determined by differences in microarray probe sequences used to detect mRNA transcripts [37] and in algorithms used for predictor genes identification. Hence, the parallel between Di Pietro's and our results is significant given that data were generated using a different set of samples and a different microarray platform, and suggests that the commonly identified set of genes may represent the most significant differences between MSI-H and MSS colon cancers (Additional file 6). Unfortunately, data sets from published reports were not available for cross confirmation of our list of predictive genes on published microarray data.

In this report, quantitative real time PCRs for differentially expressed mRNAs and Northern blot for differentially expressed miRNAs were performed to validate microarray expression data. These methods confirmed the differential expression detected by microarray-based expression methods. Additional evidence for the robustness of data was the finding that the *MLH1 *gene is among the most significantly (P < 0.001) down-regulated genes in MSI-H tumors versus the MSS cancer set (Additional file 2). Indeed, this gene represents a sort of internal control, since it is well-known that the loss of *MLH1 *function, which confers the microsatellite instability phenotype to tumor samples, is caused by transcriptional silencing due to promoter methylation in MSI tumors [5,6].

In addition to their role as discriminating markers of MSS versus MSI-H tumors, some of the gene-associated functions may possibly be involved in the different phenotypes that characterize the two types of colon cancers. In fact, analysis of the functions associated with 212 (for which annotation was present) of the 451 differentially expressed genes revealed that the most frequently associated classes were cell cycle, DNA replication, recombination, repair, gastrointestinal disease and immune response (Additional file 7), suggesting that these molecular differences may be responsible for traits that distinguish MSS and MSI-H tumors. Additional studies on the molecular and biological functions of these differentially expressed genes will be required to substantiate this hypothesis.

Among the differentially expressed genes, it is interesting to note the detection of the up-regulation of several members of the *mir-17-92 *family in the MSS colon cancers. This family includes fourteen homologous miRNAs organized in three gene clusters [38]. Our study revealed that, among these, miR-17-5p, miR-20, miR-25, miR-92-1, miR-92-2, miR-93-1 and miR-106a were significantly up-regulated in MSS versus MSI-H colon cancer. The chromosomes 13 and X gene clusters were previously found up-regulated in B-cell lymphoma [39]. It was also shown that c-MYC promotes their transcription [40] and, interestingly, enforced expression of the mir-17-92 cluster acted with c-myc to accelerate tumour development in a mouse B-cell lymphoma model [39]. In human solid tumors, the chromosome 13 mir-17-92 cluster was found up-regulated in small-cell lung cancer [41] and its ectopic over-expression enhanced lung cancer cell growth [41]. Since these data indicate that members of the mir-17-92 family can act as oncogenes to promote cell growth and inhibit apoptosis, our data suggests that up-regulation of these miRNAs may have a role in the more aggressive clinical behavior of MSS versus MSI-H tumors.

## Conclusion

This report provides the first study on microRNA expression in MSI versus MSS colon cancer. We identified microRNAs that are differentially expressed between these two classes of tumors; moreover, the addition of microRNAs in the molecular classifier improves the separation between MSI and MSS cancer samples, suggesting that the mRNA/miRNA combination could provide an improved stratification of tumor-associated characters. Interestingly, the most prominent class of differentially expressed miRNAs includes various members of the oncogenic miR-17-92 family, suggesting that these microRNAs have a role in bio-pathologic characteristics that distinguish MSS versus MSI colon cancers.

## Methods

### Colorectal cancer samples

Samples of colorectal cancer tissue and matched normal colonic mucosa were obtained from fresh surgical specimens, frozen in liquid nitrogen, and stored at -80°C. Thirty nine carcinomas, 23 microsatellite stable (MSS) and 16 with high-frequency MSI (MSI-H) were analyzed in the course of the study. The clinico-pathological features of the tumors are detailed in Table 1. MSI status was determined with a fluorescence-based PCR method, using the five markers of the panel of Bethesda (BAT25, BAT26, D2S123, D5S346, and D17S250) [42]. PCR products were run in an ABI PRISM 377 DNA sequencer (Perkin-Elmer Applied Biosystems Division, Foster City, CA) and analyzed by the GeneScan 3.1 version software (Perkin-Elmer) [43]. According to the guidelines of the Workshop of Bethesda [42], tumors showing instability at two or more loci were classified as MSI-H and tumors without instability at any locus as MSS. None of the tumors included in this study exhibited instability at a single locus (low-frequency MSI or MSI-L). All MSI-H carcinomas displayed instability at mononucleotide DNA sequences (BAT25 and BAT26 markers). Tumors were also examined for expression of the DNA mismatch repair proteins MLH1 and MSH2, using the immunohistochemical analytic procedure previously described [43]. Carcinomas showing complete loss of nuclear MLH1 or MSH2 expression were classified as MLH1 or MSH2 negative, whereas cancers with normal expression of MLH1 and MSH2 gene products were classified as MLH1 and MSH2 positive. As reported in Table 1, all MSS carcinomas demonstrated normal nuclear expression of both MLH1 and MSH2 proteins (MLH1/MSH2 positive). By contrast, of the 16 MSI-H tumors 15 were MLH1 negative and one was MSH2 negative. As expected, MSI-H carcinomas were more frequently located in the proximal colon (P < 0.001) and poorly differentiated (P < 0.001) and more often contained a mucinous component (P < 0.001) with respect to MSS tumors (Table 1). Total RNA was isolated using Trizol (Invitrogen) following manufacturer's indications. RNA integrity was assessed on a 2100 Agilent Bioanalyzer. Only samples with intact RNA were used for the gene expression analysis.

### RNA labeling and microarray hybridization

RNA labeling and hybridization on microRNA microarray chips was performed as previously described. [21,22] Briefly, 5 μg of total RNA from each sample was biotin-labeled by reverse transcription using 5' biotin end labeled random examers oligo primer. Hybridization of biotin-labeled cDNA was carried out on our miRNA microarray chip (ArrayExpress accession number: A-MEXP-86), which contains 230 human microRNA probes, in triplicate. Hybridization signals were detected by biotin binding of a Streptavidin – Alexa 647 conjugate using Axon Scanner 4000B (Axon Instrument Inc. CA).

For mRNA-chip hybridization, 5 ug of total RNA were used for the production of biotinylated cRNA. Labeled cRNA was synthesized following the Ambion cRNA Synthesis Protocol and purified using an RNeasy^® ^Kit (Qiagen). cRNA yield was quantified by measuring the UV absorbance at 260 nm. Unfragmented biotinylated cRNA (about 50 ug) was used for hybridization on custom made high density oligonucleotide arrays. The array was the human 18.5K Expression Bioarray (Compugen Human Oligo Set 1.0, http://www.labonweb.com/chips/libraries.html), which contains 18,861 probes corresponding to approximately 17,260 unique human gene clusters and 18 bacterial control probes. All probes on these microarrays are 65-mer oligonucleotides spotted by contacting technologies and covalently attached to a polymeric matrix. Microarrays were hybridized in 6 × SSPE/40% formamide using a Tecan HS4800 hybridization station at 37°C for 20 hours, post-hybridization washed in 46°C pre-warmed 0.75 × TNT (1 × TNT:0.1 M Tris-HCL, pH7.5/0.15 M NaCL/0.05% Tween-20) at 46°C for one hour, and processed using a direct detection method of the biotin-containing transcripts by a Streptavidin-Alexa647 conjugate (1:500 diluted) in TNB (0.1 M Tris-HCL, pH7.5/0.15 M NaCL/0.5% Blocking Reagent-PerkinElmer) at RT for 30 min. Post-staining washing in 1 × TNT for one hour. Processed slides were scanned using Axon 4000B scanner (Molecular Device, CA). Image were quantified by GenePix Pro 6.0 software.

### Microarray data analysis

MiRNA-chip and mRNA chip raw data were normalized separately using the GeneSpring software version 7.2 (Agilent). Both on chip and on gene median methods were used to normalize gene expression data. Microarray data were then joined into one GeneSpring genome and samples were assigned to one of two groups. The comparative analysis between MSI-H and MSS samples was carried out using the Welch's t-test and the Benjamini & Hochberg or Bonferroni (for a more stringent analysis) False Discovery Rate correction. Cluster analysis was performed using the Pearson correlation as a measure of similarity. Predictions were made using both Support Vector Machine algorithm and PAM software [44]. The Gene Ontology analysis of the gene lists of interest was generated by using the web-delivered tools of Ingenuity Pathway Analysis. Data have been submitted to Array Express (Accession number E-MEXP-326).

### Northern blot of miRNAs

RNA samples (10 μg each) were electrophoresed on 15% acrylamide, 7 M urea Criterion precasted gels (Bio-Rad, Hercules, CA) and transferred onto Hybond N+ membrane (Amersham Bioscience, Piscataway, NJ). Membranes were hybridized with oligonucleotide probes, corresponding to the complementary sequences of the following mature miRNAs: miR-25 (TCA GAC CGA GAC AAG TGC AAT G) and miR-92 (CAG GCC GGG ACA AGT GCA ATA). Probes were 5'-end labeled using the polynucleotide kinase in the presence of P32-gamma-ATP. Hybridization was performed at 37°C in 7% SDS/0.2 mol/L Na2PO4 (pH 7.0) for 16 hours. Membranes were washed at 42°C, twice with 2 × standard saline phosphate (0.18 mol/L NaCl/10 mmol/L phosphate pH 7.4), 1 mmol/L EDTA (saline-sodium phosphate-EDTA, SSPE), and 0.1% SDS and twice with 0.5 × SSPE/0.1% SDS. Northern blots were re-hybridized after stripping the oligonucleotides used as probes in boiling 0.1% SDS for 10 minutes. As a control for normalization of RNA expression levels, we hybridized blots with an oligonucleotide probe complementary to the U6 RNA (5'-GCA GGG GCC ATG CTA ATC TTC TCT GTA TCG-3').

### Quantitative Real time PCR for mRNAs

The RT reaction was perfomed using 200 ng of total RNA for each sample according to the manufacturer instructions (SuperScript First Strand Invitrogen). The Real Time reactions were performed using Taqman Gene Expression Assay on a ABI PRISM 7900HT. For each gene one set of primers and a probe were chosen from the Applied Biosystems list of TaqMan^® ^Gene Expression Assays. Hs02379661_g1 for MTA2, Hs00228732_m1 for CTCL tumor antigen, Hs00228336_m1 for C13orf18, Hs00196125_m1 for VAV3 oncogene, Hs00914163_m1 for GGH, Hs00214886_m1 for FLJ20315. Expression analysis was performed in triplicate for each sample. Expression of 18S rRNA, which displayed the most constant expression among tested housekeeping genes between different samples, was used as endogenous reference control. The fold difference for each sample was obtained using the following equation 2^-dCt^. Ct is the Threshold Cycle, the cycle number at which the fluorescence generated within a reaction crosses the threshold; dCt = Ct average sample gene - Ct average 18S.

## Competing interests

The author(s) declare that they have no competing interests.

## Authors' contributions

GL, RG, FP samples histo-pathological characterization; MF, AV, C-gL, GAC microarray preparation, hybridization and data analysis; RS, quantitative RT-PCR; CMC, MN, work planning and manuscript preparation. All authors read and approved the final manuscript.

## Supplementary Material

Additional file 1 MicroRNAs differentially expressed between MSS and MSI-H colorectal cancers at P < 0.05. List of differentially expressed microRNAs Click here for file

Additional file 2 Protein-coding genes differentially expressed between MSS and MSI-H colorectal cancers at P < 0.05. List of differentially expressed protein-coding genes Click here for file

Additional file 3 Protein-coding genes differentially expressed between MSS and MSI-H colorectal cancers with Bonferroni's correction. List of differentially expressed protein-coding genes, selected from Additional file 2 using the more stringent Bonferroni's statistical correction Click here for file

Additional file 4 Classification of tumors according to expression of microRNAs. Cluster analysis based on differentially expressed microRNAs Click here for file

Additional file 5 Classification of tumors according to expression of 72 differentially expressed mRNAs. Cluster analysis based on differentially expressed mRNAs Click here for file

Additional file 6 Differentially expressed protein-coding genes shared with Di Pietro's study. List of protein-coding genes shared by this study and Di Pietro's study Click here for file

Additional file 7 Gene Ontology classification based on Ingenuity Pathway Analysis. List of functional classes present in the differentially expressed protein-coding genes Click here for file
