# Supplementary material for: mRNA/microRNA gene expression profile in microsatellite unstable colorectal cancer
Source: Mol Cancer. 2007 Aug 23;6:54. doi: 10.1186/1476-4598-6-54 (PMC2048978; doi:10.1186/1476-4598-6-54)
Supplement: Additional file 1 — MicroRNAs differentially expressed between MSS and MSI-H colorectal cancers at P < 0.05. List of differentially expressed microRNAs [file 1476-4598-6-54-S1.pdf]

Additional file 1. MicroRNAs differentially expressed between MSS and MSI-H colorectal cancers at P<0.05

		**MSS**		**MSI-H**	
**Common**	**P-value**	**Normalized**	**StdErr Norm**	**Normalized**	**StdErr Norm**
miR-223	0.0469	0.97	0.11	1.84	0.45
miR-215	0.0234	1.42	0.19	0.84	0.14
miR-155 (BIC)	0.0234	0.97	0.03	1.29	0.13
miR-192	0.0216	1.4	0.14	0.88	0.13
miR-191	0.00474	1.28	0.09	1.00	0.05
miR-203	0.00425	1.31	0.1	0.99	0.05
miR-032	0.000648	1.17	0.04	0.91	0.02
miR-017 (miR-091)	7.39E-05	1.23	0.05	0.89	0.05
miR-025	2.14E-05	1.52	0.13	0.84	0.03
miR-106a	2.04E-05	1.29	0.07	0.92	0.03
miR-092-1	1.87E-05	1.5	0.16	0.65	0.06
miR-092-2	1.87E-05	1.6	0.20	0.64	0.06
miR-093-1	1.87E-05	1.23	0.06	0.90	0.03
miR-020	9.95E-06	1.38	0.09	0.89	0.03
