# Supplementary material for: mRNA/microRNA gene expression profile in microsatellite unstable colorectal cancer
Source: Mol Cancer. 2007 Aug 23;6:54. doi: 10.1186/1476-4598-6-54 (PMC2048978; doi:10.1186/1476-4598-6-54)
Supplement: Additional file 2 — Protein-coding genes differentially expressed between MSS and MSI-H colorectal cancers at P < 0.05. List of differentially expressed protein-coding genes [file 1476-4598-6-54-S2.pdf]

Additional file 2. Protein-coding genes differentially expressed between MSS and MSI-H colorectal cancers at P<0.05

			RATIO MSS/MSI-H	MSS		MSI-H	
**Genbank**	**Symbol**	**P-value**		**Normalized**	**StdErr Norm**	**Normalized**	**StdErr Norm**
AJ227892		4.98E-02	1.50	1.234	0.134	0.822	0.057
NM_013248	NXT1	4.96E-02	1.36	1.308	0.104	0.960	0.052
AF086257	SIAT4B	4.94E-02	1.39	1.153	0.075	0.830	0.064
AL359056	STX16	4.91E-02	1.26	1.130	0.063	0.895	0.040
NM_000946	NACA	4.91E-02	1.31	1.199	0.067	0.913	0.056
AL117560		4.91E-02	1.28	1.074	0.052	0.842	0.047
NM_002535	OAS2	4.90E-02	0.84	0.969	0.032	1.157	0.048
AB002330	SR140	4.84E-02	1.21	1.125	0.059	0.929	0.022
NM_016484	LOC51248	4.83E-02	1.52	1.225	0.116	0.806	0.073
NM_016593	CYP39A1	4.83E-02	1.24	1.144	0.066	0.919	0.029
NM_006044	HDAC6	4.83E-02	1.29	1.118	0.063	0.870	0.045
AK024322	FLJ14260	4.81E-02	1.25	1.130	0.059	0.901	0.040
NM_016008	D2LIC	4.78E-02	1.21	1.046	0.027	0.862	0.043
AF305836	DIO3OS	4.77E-02	1.31	1.088	0.062	0.829	0.049
NM_006833	COPS6	4.77E-02	1.42	1.226	0.090	0.861	0.069
AB007932	PLXNA2	4.77E-02	1.40	1.027	0.059	0.733	0.061
AK024674	DLGAP4; DAP4; SAPAP4; KIAA0964	4.77E-02	1.21	1.082	0.040	0.897	0.038
AB020660	KIAA0853	4.77E-02	1.48	1.241	0.118	0.839	0.065
AK024303		4.77E-02	2.45	2.424	0.792	0.989	0.152
AK027155		4.77E-02	1.19	1.105	0.048	0.926	0.029
NM_006834	RAB32	4.77E-02	1.86	1.171	0.141	0.628	0.101
AL050367	LOC221061	4.73E-02	1.72	1.118	0.106	0.651	0.091
AB002448		4.73E-02	1.59	1.184	0.112	0.746	0.081
AK023245	FLJ21144	4.73E-02	1.24	1.125	0.035	0.910	0.049
AK023307	SLC13A3	4.69E-02	1.14	1.075	0.038	0.939	0.018
NM_018050	FLJ10298	4.67E-02	1.91	1.414	0.161	0.741	0.128
U30828	SFRS6	4.65E-02	1.59	1.410	0.162	0.885	0.082
Y16704		4.65E-02	2.24	1.273	0.127	0.567	0.143
AF273052	C13ORF10	4.64E-02	1.63	1.228	0.129	0.753	0.084

AK026642		4.63E-02	1.96	1.265	0.160	0.645	0.115
NM_003720	DSCR2	4.63E-02	1.59	1.273	0.114	0.801	0.089
U66048		4.59E-02	1.16	1.106	0.043	0.950	0.022
AK021744		4.58E-02	1.30	1.193	0.069	0.916	0.050
NM_006020	ALKBH	4.58E-02	1.23	1.090	0.060	0.885	0.026
NM_002763	PROX1	4.58E-02	1.20	1.112	0.052	0.923	0.028
AL117461	MGC21416	4.58E-02	1.41	1.131	0.071	0.804	0.065
NM_000554	CRX	4.58E-02	1.25	1.062	0.039	0.851	0.046
NM_019020	TBC1D16	4.57E-02	1.33	1.224	0.082	0.919	0.051
AK022207	MLPH	4.57E-02	0.77	0.884	0.036	1.147	0.074
AF088033	VCIP135	4.57E-02	1.23	1.112	0.054	0.903	0.036
NM_002157	HSPE1	4.56E-02	2.26	1.355	0.226	0.599	0.133
AK021962		4.56E-02	1.26	1.092	0.056	0.863	0.042
NM_006253	PRKAB1	4.56E-02	1.62	1.258	0.122	0.776	0.088
NM_006144	GZMA	4.56E-02	0.59	0.885	0.056	1.506	0.221
AK024653	LOC254057	4.56E-02	1.24	1.122	0.063	0.907	0.027
NM_006809	TOMM34	4.56E-02	1.39	1.217	0.094	0.874	0.057
NM_000488	SERPINC1	4.56E-02	0.85	0.941	0.026	1.107	0.043
AF169796	RAD18	4.56E-02	1.17	1.089	0.036	0.933	0.030
NM_004813	PEX16	4.53E-02	1.24	1.112	0.052	0.898	0.039
NM_003240	EBAF	4.53E-02	1.91	1.572	0.256	0.823	0.113
NM_016276	SGK2	4.53E-02	1.47	1.195	0.101	0.810	0.067
NM_014479	ADAMDEC1	4.53E-02	0.80	0.978	0.037	1.216	0.062
NM_007222	ZHX1	4.53E-02	1.45	1.238	0.103	0.855	0.066
NM_000282	PCCA	4.53E-02	1.72	1.283	0.158	0.745	0.089
AK025341	FARP1	4.53E-02	1.19	1.077	0.046	0.902	0.028
NM_016500	MGC874	4.50E-02	1.43	1.180	0.087	0.828	0.064
NM_005815	ZNF443	4.49E-02	1.40	1.196	0.065	0.857	0.071
NM_005541	INPP5D	4.46E-02	1.38	1.291	0.083	0.938	0.067
L39061	TAF1B	4.46E-02	1.28	1.071	0.052	0.839	0.045
NM_006763	BTG2	4.45E-02	1.60	1.349	0.118	0.844	0.095
NM_001260	CDK8	4.45E-02	1.47	1.232	0.108	0.837	0.066
NM_006638	RNASEP1	4.45E-02	1.60	1.281	0.132	0.801	0.081
AL049266		4.45E-02	1.75	1.245	0.109	0.712	0.105
AK022414	KIAA1797	4.42E-02	1.20	1.137	0.033	0.945	0.042

AL133438	TGOLN2	4.41E-02	1.42	1.083	0.073	0.762	0.061
X98260	ZRF1	4.41E-02	1.33	1.222	0.084	0.921	0.045
NM_015384	IDN3	4.41E-02	1.27	1.093	0.049	0.858	0.047
AF086008	KIAA0146	4.41E-02	1.14	1.073	0.028	0.938	0.028
AL117477	PHF19	4.39E-02	1.28	1.114	0.057	0.868	0.047
AF271070	SLC38A1	4.39E-02	2.67	1.487	0.308	0.557	0.159
NM_012333	MYCBP	4.34E-02	1.27	1.041	0.052	0.820	0.041
U87460		4.32E-02	1.34	1.105	0.074	0.827	0.046
NM_000045	ARG1	4.25E-02	0.81	0.939	0.031	1.157	0.058
NM_002985	CCL5	4.24E-02	0.70	0.867	0.045	1.239	0.111
AF251684	TBX22	4.21E-02	0.82	0.923	0.030	1.122	0.052
NM_003932	ST13	4.20E-02	1.32	1.126	0.074	0.856	0.041
AK026416		4.14E-02	1.41	1.157	0.098	0.820	0.049
AK025359	ABTB2	4.13E-02	1.18	1.082	0.037	0.916	0.032
NM_006980	MTERF	4.13E-02	1.52	1.275	0.106	0.836	0.079
NM_015644	TTLL3	4.12E-02	1.23	1.149	0.057	0.933	0.033
AK026276	HSPBAP1	4.12E-02	1.31	1.103	0.056	0.839	0.051
AB007962	KIAA0493	4.12E-02	1.21	1.128	0.045	0.930	0.037
L32136		4.12E-02	1.49	1.190	0.087	0.797	0.074
NM_020158	RRP46	4.11E-02	1.13	1.090	0.032	0.963	0.021
NM_003004	SECTM1	4.11E-02	0.43	0.717	0.081	1.658	0.415
AF086205	TUFT1	4.11E-02	1.26	1.075	0.059	0.854	0.033
NM_003031	SIAH1	4.11E-02	1.62	1.159	0.084	0.715	0.088
NM_016261	TUBD1	4.11E-02	1.38	1.153	0.061	0.834	0.065
AK021716	FAM11B	4.11E-02	1.23	1.111	0.037	0.907	0.043
AL050353	OIP2	4.11E-02	1.27	1.153	0.062	0.906	0.041
NM_018270	C20ORF20	4.11E-02	1.36	1.271	0.095	0.931	0.051
NM_007173	SPUVE	4.11E-02	2.00	1.649	0.243	0.826	0.135
AK024611	C6ORF79	4.06E-02	1.26	1.116	0.058	0.883	0.040
D17093	MGC34680	4.06E-02	1.25	1.082	0.048	0.867	0.040
AL137473	C20ORF67; PCIF1	4.06E-02	1.22	1.097	0.045	0.896	0.037
AK022180	FLJ12118	4.03E-02	1.40	1.153	0.079	0.823	0.059
NM_017791	C14ORF58	4.03E-02	0.82	0.926	0.023	1.129	0.055
NM_006531	TG737	4.03E-02	1.49	1.202	0.110	0.809	0.061
NM_002740	PRKCI	4.02E-02	1.53	1.455	0.164	0.949	0.061

AL365512	HPS4	4.02E-02	1.32	1.135	0.070	0.857	0.047
AK023965	LOC114926	4.02E-02	1.47	1.225	0.107	0.836	0.062
AK025271	CHCHD3	4.02E-02	1.21	1.120	0.053	0.929	0.025
NM_020197	SMYD2	3.99E-02	1.40	1.175	0.083	0.837	0.059
AK000839		3.98E-02	1.46	1.278	0.124	0.877	0.050
AB033096	AARSL	3.96E-02	1.20	1.097	0.044	0.915	0.031
NM_016652	CRNKL1	3.96E-02	1.47	1.228	0.111	0.835	0.060
X97261	MT1L	3.92E-02	0.46	0.627	0.092	1.365	0.275
NM_006413	RPP30	3.90E-02	1.54	1.120	0.076	0.728	0.076
AL117601	FLJ35848	3.90E-02	1.14	1.074	0.034	0.942	0.020
NM_004344	CETN2	3.84E-02	1.53	1.251	0.106	0.818	0.076
NM_017569	P38IP	3.84E-02	1.22	1.092	0.038	0.893	0.040
NM_012483	GNLY	3.84E-02	0.82	0.976	0.038	1.188	0.049
AF161441	LOC284422	3.84E-02	1.46	1.231	0.118	0.842	0.051
NM_018372	RIF1	3.84E-02	1.75	1.285	0.135	0.735	0.096
X60155	ZNF41	3.84E-02	1.21	1.094	0.039	0.904	0.037
AK026142	ODAG	3.84E-02	1.43	1.294	0.115	0.903	0.053
AK021555		3.84E-02	1.65	1.350	0.161	0.820	0.077
NM_006596	POLQ	3.84E-02	1.52	1.191	0.085	0.784	0.077
AK024181	FLJ35155	3.84E-02	1.20	1.064	0.033	0.885	0.037
AK021907	NOL5A	3.84E-02	1.29	1.140	0.065	0.884	0.042
AK075306	TIF1	3.84E-02	1.32	1.137	0.055	0.861	0.054
NM_016617	BM-002	3.84E-02	1.52	1.205	0.100	0.794	0.072
AK025605	ZDHHC6	3.84E-02	1.30	1.142	0.052	0.878	0.052
AK055991	LAMR1	3.83E-02	1.21	1.120	0.034	0.928	0.040
L07517	MUC6	3.82E-02	1.55	1.347	0.138	0.870	0.070
AK024573	FLJ20920	3.82E-02	1.45	1.251	0.108	0.862	0.058
AF202637	PP3111	3.82E-02	1.71	1.382	0.139	0.808	0.100
NM_003173	SUV39H1	3.82E-02	1.40	1.193	0.068	0.855	0.065
AK024209		3.82E-02	1.18	1.093	0.036	0.924	0.032
NM_015969	MRPS17	3.82E-02	1.41	1.217	0.094	0.865	0.055
NM_014373	GPR160	3.82E-02	1.95	1.453	0.189	0.743	0.119
NM_016001	CGI-48	3.82E-02	1.76	1.227	0.131	0.695	0.092
NM_002450		3.82E-02	0.53	0.664	0.075	1.244	0.190
NM_015931	LOC51066	3.81E-02	1.57	1.369	0.168	0.872	0.049

AL110152	CD109	3.81E-02	0.52	0.915	0.047	1.745	0.313
NM_001427	EN2	3.81E-02	1.20	1.080	0.047	0.898	0.027
X91648	PURA	3.81E-02	1.51	1.197	0.113	0.792	0.062
AF237813	ABAT	3.81E-02	1.44	1.100	0.094	0.763	0.050
NM_001455	FOXO3A	3.81E-02	1.39	1.258	0.090	0.907	0.057
AK027156	PCSK7	3.81E-02	1.18	1.092	0.038	0.926	0.029
X64643	C6.1A	3.77E-02	1.17	1.126	0.041	0.958	0.026
AF086208	FGF2; BFGF; FGFB	3.77E-02	0.86	0.938	0.028	1.097	0.036
NM_015942	CGI-12	3.77E-02	1.70	1.290	0.139	0.757	0.088
AV704019	KCNK12	3.77E-02	0.79	0.930	0.036	1.173	0.062
NM_002777	PRTN3	3.77E-02	0.83	0.955	0.030	1.152	0.049
AB000509	TRAF5	3.70E-02	1.73	1.367	0.156	0.789	0.093
AK024462	SN	3.70E-02	0.74	0.935	0.034	1.268	0.095
BC000363	REPIN1	3.70E-02	1.18	1.070	0.030	0.904	0.034
D86978	NUP205	3.70E-02	1.70	1.201	0.111	0.705	0.088
AF086220	FLJ32130	3.62E-02	1.29	1.182	0.068	0.915	0.041
NM_012091	ADAT1	3.62E-02	1.23	1.063	0.040	0.863	0.038
NM_007027	TOPBP1	3.54E-02	1.20	1.133	0.043	0.942	0.034
AK023339	MGC40214	3.49E-02	1.38	1.175	0.081	0.852	0.052
NM_016071	MRPS33	3.46E-02	1.78	1.089	0.086	0.613	0.089
AJ272267	CHDH	3.42E-02	1.38	1.200	0.077	0.869	0.057
AK025238	LRRC5	3.42E-02	1.18	1.072	0.033	0.912	0.030
AK023574	SLC40A1	3.42E-02	1.43	1.277	0.109	0.894	0.051
AK026225	PLD1	3.40E-02	1.38	1.151	0.061	0.837	0.060
NM_002465	MYBPC1	3.39E-02	0.81	0.927	0.029	1.142	0.054
U79246	FLJ22624	3.39E-02	1.40	1.241	0.098	0.886	0.049
AL359596	DKFZP547P055	3.39E-02	1.39	1.137	0.071	0.815	0.057
AF088004	CACNA1D	3.38E-02	1.52	1.281	0.116	0.841	0.068
NM_014052	YWHAB	3.38E-02	2.05	1.381	0.202	0.674	0.110
NM_016100	NAT5	3.38E-02	1.21	1.128	0.045	0.934	0.033
AL359585		3.37E-02	1.62	1.316	0.123	0.811	0.084
AJ002969		3.35E-02	1.35	1.104	0.050	0.819	0.056
AL360145		3.35E-02	1.17	1.086	0.037	0.924	0.027
BC036692	LOC283824	3.24E-02	1.26	1.053	0.042	0.834	0.042

NM_006698	BLCAP	3.24E-02	1.47	1.263	0.111	0.858	0.058
NM_017948	NOL8	3.24E-02	1.65	1.241	0.105	0.754	0.086
AF272890	ADRB1	3.24E-02	1.23	1.104	0.043	0.895	0.039
D87682	KIAA0241	3.24E-02	1.21	1.087	0.037	0.899	0.036
AK022337	NEBL	3.23E-02	1.27	1.209	0.063	0.948	0.041
AL050090	MYRIP	3.23E-02	2.40	1.361	0.230	0.566	0.124
NM_018318	FLJ11088	3.23E-02	1.70	1.289	0.123	0.756	0.090
NM_001000	RPL39	3.23E-02	1.75	1.219	0.092	0.696	0.096
AF086558	KIAA0350	3.16E-02	1.25	1.149	0.052	0.919	0.039
NM_003716	CADPS	3.16E-02	1.26	1.193	0.065	0.946	0.032
AJ250475	UCC1	3.11E-02	2.00	1.698	0.274	0.848	0.116
M96577	E2F1	3.11E-02	1.56	1.320	0.109	0.849	0.079
NM_006089	SCML2	3.11E-02	2.27	1.715	0.207	0.754	0.163
NM_014835	OSBPL2	3.10E-02	1.64	1.304	0.131	0.793	0.080
NM_017812	CHCHD3	3.10E-02	1.80	1.362	0.176	0.756	0.087
AK025445		3.07E-02	1.17	1.060	0.034	0.906	0.026
AF181994	FANCF	3.04E-02	1.48	1.337	0.109	0.905	0.066
AK023620		3.04E-02	1.50	1.150	0.078	0.769	0.068
NM_015725	RDH8	3.04E-02	1.59	1.307	0.129	0.824	0.072
AK022722	C14ORF127	3.01E-02	1.41	1.225	0.097	0.866	0.048
NM_015904	EIF5B	3.00E-02	1.46	1.207	0.083	0.828	0.065
NM_002844	PTPRK	3.00E-02	1.67	1.165	0.098	0.697	0.081
NM_007127	VIL1	3.00E-02	2.87	1.294	0.210	0.451	0.138
AK024379	CDC2L5	3.00E-02	1.21	1.148	0.043	0.945	0.036
AK023296	XRCC5	2.99E-02	1.33	1.181	0.073	0.888	0.043
NM_006648	PRKWNK2	2.95E-02	1.79	1.341	0.153	0.749	0.092
NM_017936	KIAA2010	2.95E-02	1.23	1.054	0.033	0.858	0.039
NM_013974	DDAH2	2.95E-02	1.23	1.164	0.047	0.944	0.038
AK024433	MRPS25	2.95E-02	1.50	1.198	0.094	0.798	0.065
AK023031	FLJ12969	2.95E-02	1.32	1.106	0.060	0.836	0.045
NM_014397	NEK6	2.94E-02	1.58	1.234	0.104	0.779	0.075
NM_003079	SMARCE1	2.84E-02	1.20	1.113	0.042	0.931	0.029
AF117233	MKRN1	2.83E-02	1.57	1.222	0.095	0.779	0.075
AK024275	FLJ14213	2.83E-02	1.77	1.237	0.104	0.697	0.094
AK021523	KIAA1068	2.79E-02	1.30	1.129	0.054	0.866	0.046

NM_014166	VDRIP	2.79E-02	1.61	1.308	0.123	0.810	0.077
AF086344	HIWI2	2.79E-02	1.65	1.245	0.106	0.755	0.082
AK024292	LOC151648	2.79E-02	1.23	1.143	0.058	0.926	0.023
AF147408	RSC1A1; RS1	2.79E-02	1.51	1.324	0.120	0.878	0.061
D17267	CSNK2A2	2.79E-02	1.63	1.186	0.104	0.728	0.075
NM_002128	HMGB1	2.79E-02	1.54	1.320	0.127	0.855	0.064
AK026295		2.78E-02	2.00	1.297	0.175	0.647	0.097
NM_004294	MTRF1	2.78E-02	1.25	1.135	0.060	0.908	0.026
AF075017		2.78E-02	1.23	1.112	0.038	0.906	0.038
AL117464		2.78E-02	1.16	1.051	0.033	0.904	0.023
NM_006024	TAX1BP1	2.78E-02	1.61	1.162	0.111	0.723	0.066
NM_000057	BLM	2.78E-02	1.18	1.093	0.037	0.924	0.028
AK026412	DHX35	2.78E-02	1.23	1.164	0.052	0.949	0.031
NM_013995	LAMP2	2.78E-02	1.90	1.274	0.134	0.671	0.099
NM_016542	MST4	2.78E-02	1.49	1.202	0.097	0.808	0.060
NM_018122	FLJ10514	2.78E-02	1.29	1.115	0.057	0.865	0.040
BC013695	ZNF313	2.78E-02	1.75	1.452	0.137	0.830	0.103
AK023104		2.78E-02	1.32	1.110	0.047	0.839	0.051
NM_006304	SHFM1	2.78E-02	1.75	1.218	0.129	0.695	0.081
U88897		2.77E-02	1.23	1.087	0.048	0.881	0.031
AF101051	CLDN1	2.77E-02	2.39	1.679	0.303	0.702	0.137
NM_004586	RPS6KA3	2.77E-02	1.40	1.204	0.079	0.858	0.056
AL049974		2.77E-02	1.27	1.106	0.059	0.868	0.032
AK056222		2.75E-02	1.30	1.169	0.070	0.897	0.036
NM_006176	NRGN	2.72E-02	0.69	0.840	0.049	1.222	0.099
AK022223	FLJ22955	2.71E-02	1.32	1.191	0.068	0.900	0.045
AK024522	GPCR5A	2.71E-02	2.04	1.392	0.210	0.684	0.098
AF085951	MCSC	2.71E-02	1.43	1.141	0.062	0.796	0.062
NM_019005	FLJ20323	2.68E-02	1.52	1.224	0.100	0.806	0.064
AK026277	FLJ22624	2.63E-02	1.80	1.326	0.124	0.738	0.097
NM_018173	FLJ10665	2.58E-02	1.37	1.127	0.053	0.824	0.055
AL110262	NCBP2	2.49E-02	1.63	1.222	0.123	0.751	0.067
AF218021		2.48E-02	1.28	1.206	0.068	0.942	0.031
NM_001782	CD72	2.42E-02	0.83	0.953	0.028	1.143	0.042
S81973	PHC3	2.41E-02	1.20	1.116	0.048	0.928	0.021

NM_012415	RAD54B	2.40E-02	1.34	1.216	0.069	0.906	0.049
AF086155	MGC2776	2.32E-02	1.41	1.157	0.070	0.821	0.055
AK024399		2.30E-02	1.26	1.048	0.035	0.829	0.041
NM_005830	MRPS31	2.30E-02	1.84	1.177	0.134	0.639	0.079
NM_003074	SMARCC1	2.30E-02	1.24	1.095	0.048	0.884	0.031
NM_003568	ANXA9	2.30E-02	1.25	1.092	0.040	0.873	0.039
AK023408	C20ORF172	2.30E-02	1.62	1.327	0.143	0.818	0.063
AB037795	KIAA1374	2.30E-02	1.41	1.207	0.078	0.854	0.056
NM_005855	RAMP1	2.30E-02	0.67	0.968	0.033	1.442	0.132
NM_001948	DUT	2.28E-02	1.36	1.187	0.071	0.872	0.048
AK025766	BRI3BP	2.28E-02	2.38	1.336	0.190	0.561	0.115
NM_014936	ENPP4	2.28E-02	0.81	0.905	0.031	1.112	0.045
NM_000151	G6PC	2.28E-02	1.65	1.238	0.105	0.748	0.078
NM_018278		2.28E-02	1.19	1.098	0.040	0.925	0.024
AF069307	SLC5A6	2.28E-02	1.36	1.176	0.074	0.863	0.046
NM_003348	UBE2N	2.27E-02	1.21	1.104	0.040	0.911	0.031
NM_001951	E2F5	2.26E-02	2.05	1.326	0.152	0.646	0.104
NM_001445	FABP6	2.26E-02	1.43	1.239	0.088	0.866	0.054
AK025546		2.23E-02	1.27	1.127	0.061	0.889	0.025
AB037756	CHD6	2.23E-02	1.52	1.360	0.113	0.895	0.067
AJ009817	AMMECR1	2.23E-02	1.23	1.077	0.032	0.874	0.038
AK025686	MGC21654	2.23E-02	1.20	1.127	0.044	0.936	0.027
AK000053	MCLC	2.23E-02	1.29	1.209	0.074	0.936	0.021
NM_003534	HIST1H3G	2.23E-02	1.54	1.147	0.078	0.747	0.066
NM_004067	CHN2	2.22E-02	1.68	1.338	0.171	0.798	0.048
AK000548	C20ORF52	2.22E-02	1.97	1.253	0.126	0.636	0.097
NM_152747	DKFZP586I1420	2.17E-02	1.28	1.090	0.048	0.854	0.039
AK022097	DOCK7	2.17E-02	1.41	1.257	0.103	0.890	0.034
AF147347	DACH1	2.17E-02	1.36	1.156	0.198	0.851	0.039
NM_002296	LBR	2.17E-02	1.95	1.069	0.102	0.548	0.083
NM_001208	BTF3L1; OPG; OCIF; HUMBTFB; TNFRSF11B	2.17E-02	1.43	1.220	0.076	0.853	0.059
AK022122	MTBP; MDM2BP	2.11E-02	1.23	1.183	0.049	0.958	0.033
NM_002853	RAD1	2.11E-02	1.56	1.298	0.123	0.833	0.058

AL157440	C10ORF116	2.11E-02	1.27	1.101	0.040	0.867	0.042
NM_018455	BM039	2.10E-02	1.60	1.231	0.098	0.770	0.072
NM_006421	BIG1	2.10E-02	1.68	1.335	0.124	0.792	0.081
AF070559	LOC93081	2.09E-02	1.40	1.243	0.087	0.888	0.047
AF009308	SEMA5A	2.09E-02	1.46	1.125	0.063	0.769	0.060
AK022164	C9ORF5	2.09E-02	1.33	1.177	0.073	0.885	0.035
NM_006420	ARFGEF2	2.09E-02	1.38	1.201	0.068	0.868	0.053
NM_001178	ARNTL	2.06E-02	1.27	1.095	0.044	0.864	0.039
NM_017798	C20ORF21	2.06E-02	1.81	1.321	0.145	0.732	0.083
AF009316	SEMA5A	2.05E-02	1.19	1.114	0.041	0.937	0.023
NM_017952	FLJ20758	2.04E-02	1.44	1.328	0.103	0.922	0.051
AK025259	MYO15B	1.98E-02	1.45	1.255	0.094	0.867	0.052
AF298880	XPO5	1.98E-02	1.61	1.349	0.231	0.836	0.072
AK001279		1.95E-02	1.62	1.281	0.125	0.790	0.064
D00265	CYCS	1.95E-02	1.62	1.131	0.095	0.697	0.065
NM_012260	HPCL2	1.93E-02	1.42	1.192	0.070	0.839	0.057
AF086341		1.91E-02	1.70	1.314	0.115	0.772	0.082
AK023228	ADAMTS20	1.91E-02	1.35	1.121	0.055	0.829	0.048
AF231997	GAS7	1.91E-02	1.22	1.094	0.045	0.896	0.027
NM_014553	TFCP2L1	1.91E-02	1.43	1.157	0.077	0.808	0.051
AF052109		1.85E-02	0.81	0.926	0.020	1.140	0.050
NM_005520	HNRPH1	1.84E-02	0.82	0.970	0.027	1.180	0.046
AK022111	BCL2	1.84E-02	0.82	0.964	0.028	1.181	0.048
X96644	RNU45A; U45A	1.84E-02	1.33	1.185	0.055	0.893	0.048
NM_004626	WNT11	1.78E-02	1.46	1.284	0.099	0.882	0.050
NM_004507	HUS1	1.78E-02	1.25	1.166	0.049	0.930	0.035
AK025173	EPHB2	1.76E-02	1.37	1.145	0.057	0.834	0.051
NM_001316	CSE1L	1.74E-02	2.26	1.452	0.219	0.642	0.105
NM_007052	NOX1	1.74E-02	1.34	1.255	0.079	0.936	0.035
AK026497	YY1	1.73E-02	1.49	1.106	0.065	0.740	0.059
NM_006721	ADK	1.69E-02	1.22	1.115	0.039	0.912	0.032
AF131784	RAB27B	1.69E-02	0.45	0.766	0.071	1.700	0.312
NM_001363	DKC1	1.65E-02	2.31	1.530	0.207	0.662	0.119
AK023665	PRLR	1.65E-02	1.38	1.198	0.068	0.867	0.050
NM_013435	RAX	1.65E-02	1.77	1.414	0.115	0.797	0.094

NM_002097	GTF3A	1.65E-02	1.64	1.351	0.121	0.825	0.072
NM_016004	C20ORF9	1.65E-02	1.40	1.302	0.085	0.933	0.049
AK022587	FLJ12525	1.65E-02	1.42	1.146	0.057	0.806	0.056
AB007976	KIAA0507	1.63E-02	1.45	1.288	0.089	0.887	0.056
NM_005256	GAS2	1.63E-02	1.41	1.297	0.096	0.923	0.037
NM_006346	PIBF1	1.60E-02	1.39	1.149	0.067	0.829	0.047
AK024716	RRS1	1.59E-02	1.63	1.279	0.098	0.786	0.074
NM_001265	CDX2	1.58E-02	2.22	1.115	0.107	0.501	0.090
AF070580		1.58E-02	1.22	1.093	0.034	0.897	0.033
NM_000341	SLC3A1	1.58E-02	1.56	1.333	0.127	0.857	0.050
AK023086		1.58E-02	1.39	1.228	0.067	0.882	0.053
AK023018		1.55E-02	1.27	1.147	0.048	0.905	0.036
NM_002083	GPX2	1.55E-02	3.00	1.628	0.259	0.542	0.144
NM_000845	GRM8	1.55E-02	1.33	1.222	0.072	0.916	0.036
AK022400	PNUTL2	1.53E-02	1.25	1.108	0.043	0.886	0.034
NM_006456	SIAT7B	1.52E-02	0.80	0.935	0.018	1.164	0.052
AF147339	FLJ13263	1.52E-02	1.32	1.123	0.046	0.849	0.045
AL080060		1.50E-02	1.64	1.262	0.096	0.770	0.073
NM_015922	H105E3	1.46E-02	1.26	1.134	0.051	0.902	0.030
NM_004257	TGFBRAP1	1.46E-02	1.29	1.128	0.041	0.876	0.042
J03048	HPX	1.44E-02	0.74	0.900	0.031	1.224	0.076
AK027088	STK4; KRS2; MST1	1.40E-02	1.60	1.311	0.102	0.818	0.070
AK027208	HSPC065	1.40E-02	1.28	1.165	0.059	0.912	0.028
NM_001432	EREG	1.38E-02	3.83	2.700	1.018	0.705	0.175
AK022667	FLJ11712	1.38E-02	1.81	1.415	0.156	0.782	0.079
NM_003688	CASK	1.38E-02	1.98	1.227	0.112	0.621	0.087
NM_013296	GPSM2	1.33E-02	1.82	1.678	0.217	0.924	0.073
AB056722	APCDD1	1.31E-02	3.10	2.208	0.534	0.713	0.157
NM_014665	LRRC14; KIAA0014	1.29E-02	1.40	1.158	0.069	0.827	0.045
NM_001184	ATR	1.28E-02	1.51	1.248	0.088	0.829	0.057
NM_017956	FLJ20772	1.28E-02	1.77	1.260	0.107	0.711	0.078
NM_004961	GABRE	1.24E-02	1.48	1.289	0.102	0.868	0.047
AL050027	NAALADL2	1.22E-02	1.25	1.149	0.049	0.920	0.028
NM_019847		1.22E-02	1.45	1.228	0.083	0.850	0.049
AK021963	STK24	1.14E-02	1.41	1.209	0.077	0.859	0.044

NM_004963	GUCY2C	1.13E-02	3.05	1.324	0.197	0.434	0.111
AF147414	ANKRD6	1.12E-02	1.24	1.118	0.041	0.902	0.031
AK026927	MGC5297	1.12E-02	1.50	1.186	0.075	0.792	0.056
AL137343	FAM84A	1.07E-02	3.08	1.422	0.187	0.461	0.122
AK022745		1.07E-02	1.72	1.345	0.143	0.782	0.061
AK025586	SPATA13	1.05E-02	1.55	1.305	0.097	0.844	0.060
NM_002848	PTPRO	1.05E-02	1.51	1.376	0.124	0.914	0.025
AF286095	IL22RA1	1.02E-02	1.52	1.243	0.101	0.819	0.046
NM_014252	SLC25A15	9.90E-03	1.65	1.368	0.114	0.829	0.070
NM_018140	FLJ10565	9.71E-03	1.84	1.392	0.147	0.755	0.077
NM_020202	NIT2	9.54E-03	1.85	1.383	0.130	0.746	0.083
NM_007019	UBE2C	9.45E-03	2.39	1.480	0.178	0.618	0.109
AK025798	LOC203547	9.45E-03	2.01	1.288	0.099	0.641	0.090
NM_017453	STAU	9.40E-03	1.90	1.377	0.131	0.726	0.085
AL050021	SLC7A1	9.40E-03	1.86	1.305	0.135	0.701	0.074
NM_001637	AOAH	9.27E-03	1.70	1.292	0.125	0.762	0.060
AF086310		9.27E-03	1.55	1.237	0.080	0.797	0.061
NM_003137	SRPK1	9.27E-03	1.75	1.343	0.134	0.765	0.068
NM_013275	ANKRD11	8.91E-03	1.26	1.116	0.042	0.882	0.033
NM_000366	TPM1	8.80E-03	1.49	1.247	0.085	0.839	0.051
AL137703	PRKCBP1	8.80E-03	1.44	1.238	0.075	0.861	0.049
NM_001505	GPR30	8.80E-03	0.77	0.932	0.034	1.207	0.054
NM_007238	PXMP4	8.80E-03	1.51	1.211	0.093	0.800	0.046
AB032991	NDFIP2	8.80E-03	1.92	1.303	0.131	0.678	0.079
AK022842	ZDHHC23	8.80E-03	1.70	1.248	0.115	0.736	0.061
AK023458	SIAH1	8.80E-03	1.22	1.104	0.040	0.903	0.025
NM_005170	ASCL2	8.77E-03	1.57	1.447	0.127	0.921	0.052
NM_004183	VMD2	8.73E-03	4.82	1.854	0.343	0.385	0.166
AL117665	GEMIN5	8.41E-03	1.23	1.085	0.034	0.884	0.029
NM_003185	TAF4	8.36E-03	1.47	1.190	0.070	0.810	0.052
X74804		8.36E-03	1.46	1.112	0.052	0.764	0.052
AK023449	AREG	8.08E-03	1.58	1.358	0.125	0.860	0.042
AK021793	LOC286025	7.82E-03	1.22	1.135	0.035	0.928	0.029
NM_017583	TRIM44	7.80E-03	1.34	1.210	0.061	0.904	0.037
AK023952		7.73E-03	1.48	1.099	0.053	0.740	0.052

AK025297	GABRB1	7.73E-03	1.53	1.322	0.114	0.865	0.035
AK025562	CLCN5	7.42E-03	1.57	1.335	0.096	0.851	0.061
AK000246		7.42E-03	1.46	1.118	0.062	0.766	0.048
NM_005093	CBFA2T2	6.91E-03	1.22	1.092	0.032	0.898	0.028
AK025523	C2ORF26	6.19E-03	2.04	1.394	0.178	0.684	0.071
NM_004392	DACH	6.19E-03	1.31	1.192	0.061	0.911	0.025
D87455	KIAA0266	6.19E-03	1.67	1.428	0.138	0.854	0.054
AK023112		5.99E-03	1.58	1.141	0.081	0.722	0.051
NM_006113	VAV3	5.98E-03	2.88	1.533	0.205	0.533	0.111
AF086017	LOC286148	5.93E-03	1.73	1.291	0.105	0.748	0.066
AK022976	DKFZP762P2111	5.93E-03	1.66	1.238	0.097	0.747	0.059
NM_005953	MT2A	5.93E-03	0.36	0.610	0.087	1.688	0.323
AK023542		5.90E-03	1.41	1.161	0.062	0.825	0.042
NM_007271	STK38	5.65E-03	1.23	1.096	0.035	0.890	0.028
NM_005800	D13S106E	5.62E-03	1.58	1.387	0.119	0.876	0.045
NM_015594	DKFZP434O047	5.38E-03	1.39	1.119	0.053	0.805	0.042
Y11167	RPL30	5.19E-03	1.45	1.220	0.066	0.839	0.049
AK026372	KIAA1718	5.09E-03	1.67	1.236	0.078	0.740	0.064
AK021754		5.01E-03	1.63	1.464	0.136	0.898	0.045
NM_003129	SQLE	5.01E-03	2.05	1.514	0.192	0.740	0.073
AF075083	KRT23	5.01E-03	1.38	1.207	0.072	0.875	0.029
NM_003404	YWHAB	4.78E-03	1.59	1.212	0.080	0.762	0.055
NM_000272	NPHP1	4.63E-03	1.26	1.139	0.048	0.901	0.021
AL137559	SYT7	4.61E-03	1.64	1.273	0.107	0.778	0.049
AL359055		4.18E-03	1.75	1.403	0.132	0.802	0.060
NM_016470	C20ORF111	3.88E-03	1.47	1.235	0.084	0.841	0.035
AK022995	LNX2	3.88E-03	2.24	1.493	0.171	0.666	0.087
AL110136		3.88E-03	1.35	1.214	0.068	0.897	0.023
NM_006978	ZNF183	3.88E-03	1.81	1.306	0.104	0.720	0.068
NM_003983	SLC7A6	3.88E-03	1.40	1.212	0.053	0.865	0.045
AK024240	C13ORF25	3.88E-03	1.56	1.360	0.103	0.871	0.047
NM_017726	PPP1R14D	3.88E-03	2.47	1.256	0.099	0.509	0.084
Z34290	RNU20	3.88E-03	1.31	1.146	0.054	0.878	0.022
NM_003740	KCNK5	3.88E-03	1.48	1.147	0.066	0.775	0.044
AK024761	CTNNBL1	3.88E-03	1.63	1.369	0.111	0.841	0.053

AL110283	FLJ21865	3.88E-03	1.40	1.161	0.065	0.832	0.032
AK025063	NSE1	3.51E-03	1.52	1.286	0.088	0.843	0.045
NM_003212	TDGF1	3.37E-03	1.78	1.445	0.141	0.813	0.056
AL137442	C20ORF177	3.37E-03	1.61	1.297	0.108	0.804	0.042
AK025130	FLJ21477	3.16E-03	2.16	1.334	0.168	0.618	0.064
AK024175	PDCD6	3.16E-03	1.71	1.362	0.121	0.799	0.051
NM_003918	GYG2	2.81E-03	1.33	1.088	0.039	0.817	0.035
M65066	PRKAR1B	2.81E-03	1.40	1.210	0.067	0.866	0.032
AK021595	CBFA2T2	2.81E-03	1.27	1.162	0.048	0.914	0.020
AK025683	FARP1	2.81E-03	1.57	1.202	0.073	0.767	0.050
NM_000492	CFTR	2.72E-03	1.51	1.351	0.092	0.894	0.040
NM_000687	AHCY	2.72E-03	2.32	1.443	0.166	0.623	0.080
AF273051	SE57-1	2.49E-03	2.53	2.153	0.411	0.851	0.030
NM_016234	ACSL5	2.49E-03	2.27	1.391	0.150	0.612	0.076
AL122043	DKFZP566G1424	2.26E-03	2.25	1.511	0.190	0.670	0.072
NM_006558	KHDRBS3	2.13E-03	2.46	1.455	0.171	0.590	0.081
NM_012384	GMEB2	2.13E-03	1.34	1.158	0.047	0.866	0.032
NM_016328	GTF2IRD1	2.13E-03	1.63	1.445	0.102	0.885	0.056
AK024196		1.91E-03	1.81	1.229	0.094	0.678	0.056
AK025036	DUSP18	1.70E-03	1.64	1.301	0.100	0.795	0.041
AK000532	LOC152195	1.63E-03	1.61	1.247	0.080	0.776	0.047
AF056434		1.41E-03	1.87	1.409	0.123	0.755	0.059
AF097025	NFS1	1.20E-03	1.79	1.452	0.119	0.813	0.056
L38517	IHH	1.04E-03	1.45	1.283	0.071	0.888	0.032
AK025775	C20ORF11	1.04E-03	1.89	1.380	0.135	0.729	0.048
NM_001657	AREG	1.04E-03	3.24	2.058	0.416	0.635	0.084
AK025225	NGEF	1.04E-03	1.62	1.297	0.092	0.802	0.040
NM_012413	QPCT	9.35E-04	1.71	1.481	0.122	0.869	0.041
AK026811	FLJ23053	9.08E-04	1.98	1.420	0.121	0.718	0.062
NM_000249	MLH1	8.73E-04	1.46	1.202	0.057	0.825	0.037
AB033045	KIAA1219	8.11E-04	1.57	1.283	0.076	0.816	0.042
NM_004485	GNG4	6.47E-04	1.67	1.412	0.104	0.848	0.040
NM_018267	H2AFJ	6.47E-04	1.71	1.399	0.110	0.816	0.039
AF086407	NSE1	5.74E-04	2.12	1.413	0.153	0.666	0.048
AK000276	NKD1	4.55E-04	2.90	2.339	0.409	0.807	0.045

NM_003878	GGH	7.97E-05	3.20	1.930	0.288	0.604	0.068
AK025215	C13ORF18	4.40E-05	3.19	2.261	0.320	0.708	0.077
NM_002657	PLAGL2	2.91E-05	1.68	1.318	0.078	0.782	0.033
NM_017763	FLJ20315	2.91E-05	3.47	2.007	0.256	0.578	0.070
NM_004693	K6HF	2.91E-05	1.52	1.223	0.057	0.804	0.028
