# Supplementary material for: mRNA/microRNA gene expression profile in microsatellite unstable colorectal cancer
Source: Mol Cancer. 2007 Aug 23;6:54. doi: 10.1186/1476-4598-6-54 (PMC2048978; doi:10.1186/1476-4598-6-54)
Supplement: Additional file 3 — Protein-coding genes differentially expressed between MSS and MSI-H colorectal cancers with Bonferroni's correction. List of differentially expressed protein-coding genes, selected from Additional file 2 using the more stringent Bonferroni's statistical correction [file 1476-4598-6-54-S3.pdf]

Additional file 3. Protein-coding genes differentially expressed between MSS and MSI-H colorectal cancers with Bonferroni's correction.

Genbank	Symbol		MSS		MSI-H	
**Systematic**	**Common**	**P-value**	**Normalized**	**StdErr Norm**	**Normalized**	**StdErr Norm**
AK022745	cDNA FLJ12683	4.7E-02	0.79	0.06	1.42	0.15
AL137343	FAM84A	4.7E-02	0.45	0.12	1.51	0.2
AK025586	SPATA13	4.5E-02	0.87	0.06	1.34	0.1
NM_014252	SLC25A15	4.1E-02	0.84	0.07	1.42	0.12
NM_018140	FLJ10565	4.0E-02	0.76	0.07	1.44	0.16
NM_020202	NIT2	3.9E-02	0.75	0.07	1.43	0.14
NM_007019	UBE2C	3.8E-02	0.65	0.1	1.58	0.19
AK025798	LOC203547	3.8E-02	0.69	0.07	1.31	0.11
NM_017453	STAU	3.7E-02	0.78	0.07	1.44	0.14
AL050021	SLC7A1	3.7E-02	0.73	0.08	1.39	0.14
NM_001637	AOAH	3.6E-02	0.76	0.07	1.35	0.13
NM_003137	SRPK1	3.6E-02	0.74	0.08	1.41	0.14
AF086310	ZD51F08	3.5E-02	0.8	0.07	1.28	0.08
AB032991	NDFIP2	3.2E-02	0.71	0.08	1.39	0.13
AK022842	ZDHHC23	3.2E-02	0.76	0.06	1.3	0.12
NM_007238	PXMP4	3.1E-02	0.8	0.04	1.26	0.1
NM_005170	ASCL2	3.0E-02	0.89	0.05	1.5	0.14
NM_004183	VMD2	3.0E-02	0.38	0.22	2.09	0.34
AK023449	AREG	2.6E-02	0.87	0.04	1.37	0.14
AK025297	GABRB1	2.4E-02	0.89	0.04	1.35	0.12
AK025562	CLCN5	2.2E-02	0.85	0.05	1.38	0.1
AK025523	FLJ21870	1.8E-02	0.75	0.07	1.44	0.2
D87455	KIAA0266	1.8E-02	0.89	0.04	1.47	0.15
AK023112	cDNA FLJ13050	1.7E-02	0.74	0.04	1.18	0.09
NM_006113	VAV3	1.6E-02	0.47	0.07	1.62	0.22
AK022976	DKFZP762P2111	1.6E-02	0.76	0.06	1.3	0.1
AF086017	LOC286148	1.6E-02	0.78	0.06	1.32	0.11
NM_005953	MT2A	1.6E-02	1.92	0.24	0.57	0.08
NM_005800	D13S106E	1.4E-02	0.87	0.05	1.42	0.13
AK026372	KIAA1718	1.2E-02	0.72	0.06	1.27	0.08
AK021754	FLJ11692	1.2E-02	0.92	0.05	1.51	0.15
NM_003129	SQLE	1.1E-02	0.77	0.07	1.61	0.21
NM_003404	YWHAB	1.1E-02	0.79	0.06	1.26	0.08
AL137559	SYT7	9.8E-03	0.8	0.06	1.3	0.12
AL359055	EUROIMAGE 2344436	8.7E-03	0.83	0.06	1.45	0.14
AK024761	CTNNBL1	7.9E-03	0.87	0.06	1.41	0.12
NM_006978	ZNF183	7.9E-03	0.73	0.06	1.33	0.11
AK022995	LNX2	7.8E-03	0.72	0.09	1.58	0.18
NM_017726	PPP1R14D	7.0E-03	0.52	0.09	1.31	0.1
AK024240	C13ORF25	6.8E-03	0.87	0.05	1.4	0.11
AL137442	C20ORF177	5.2E-03	0.81	0.04	1.34	0.11
NM_003212	TDGF1	5.2E-03	0.76	0.03	1.49	0.15
AK024175	PDCD6	4.6E-03	0.81	0.05	1.36	0.13

AK025130	FLJ21477	4.6E-03	0.63	0.07	1.34	0.18
AK025683	FARP1	3.8E-03	0.78	0.05	1.24	0.07
NM_000492	CFTR	3.3E-03	0.89	0.04	1.39	0.1
NM_000687	AHCY	3.3E-03	0.67	0.08	1.56	0.17
NM_016234	ACSL5	2.9E-03	0.59	0.08	1.41	0.16
AF273051	SE57-1	2.8E-03	0.84	0.03	2.21	0.46
AL122043	DKFZP566G1424	2.4E-03	0.71	0.07	1.57	0.21
NM_006558	KHDRBS3	2.2E-03	0.59	0.08	1.55	0.18
NM_016328	GTF2IRD1	2.1E-03	0.87	0.05	1.49	0.11
AK024196	FLJ14134	1.7E-03	0.69	0.06	1.26	0.1
AK025036	DUSP18	1.5E-03	0.81	0.04	1.33	0.11
AK000532	LOC152195	1.3E-03	0.77	0.04	1.29	0.08
AF056434	FBD8 Cri-du-chat	1.1E-03	0.77	0.06	1.44	0.13
AF097025	NFS1	8.8E-04	0.82	0.05	1.49	0.13
NM_001657	AREG	7.2E-04	0.69	0.1	2.1	0.47
AK025775	C20ORF11	7.0E-04	0.78	0.04	1.44	0.14
AK025225	NGEF	6.3E-04	0.87	0.08	1.35	0.1
NM_012413	QPCT	5.0E-04	0.89	0.04	1.53	0.13
AK026811	FLJ23053	4.4E-04	0.77	0.06	1.45	0.13
AB033045	KIAA1219	3.3E-04	0.83	0.04	1.33	0.07
NM_004485	GNG4	2.4E-04	0.81	0.03	1.46	0.11
NM_018267	H2AFJ	2.3E-04	0.92	0.12	1.43	0.11
AF086407	NSE1	1.6E-04	0.67	0.05	1.47	0.17
AK000276	NKD1	1.1E-04	0.77	0.03	2.47	0.45
NM_003878	GGH	1.6E-05	0.61	0.06	2.1	0.3
AK025215	C13ORF18	7.2E-06	0.66	0.05	2.41	0.35
NM_002657	PLAGL2	3.6E-06	0.76	0.03	1.37	0.08
NM_004693	K6HF	3.3E-06	0.8	0.03	1.24	0.06
NM_017763	FLJ20315	1.4E-06	0.57	0.06	2.1	0.28
