# Supplementary material for: mRNA/microRNA gene expression profile in microsatellite unstable colorectal cancer
Source: Mol Cancer. 2007 Aug 23;6:54. doi: 10.1186/1476-4598-6-54 (PMC2048978; doi:10.1186/1476-4598-6-54)
Supplement: Additional file 4 — Classification of tumors according to expression of microRNAs. Cluster analysis based on differentially expressed microRNAs [file 1476-4598-6-54-S4.pdf]

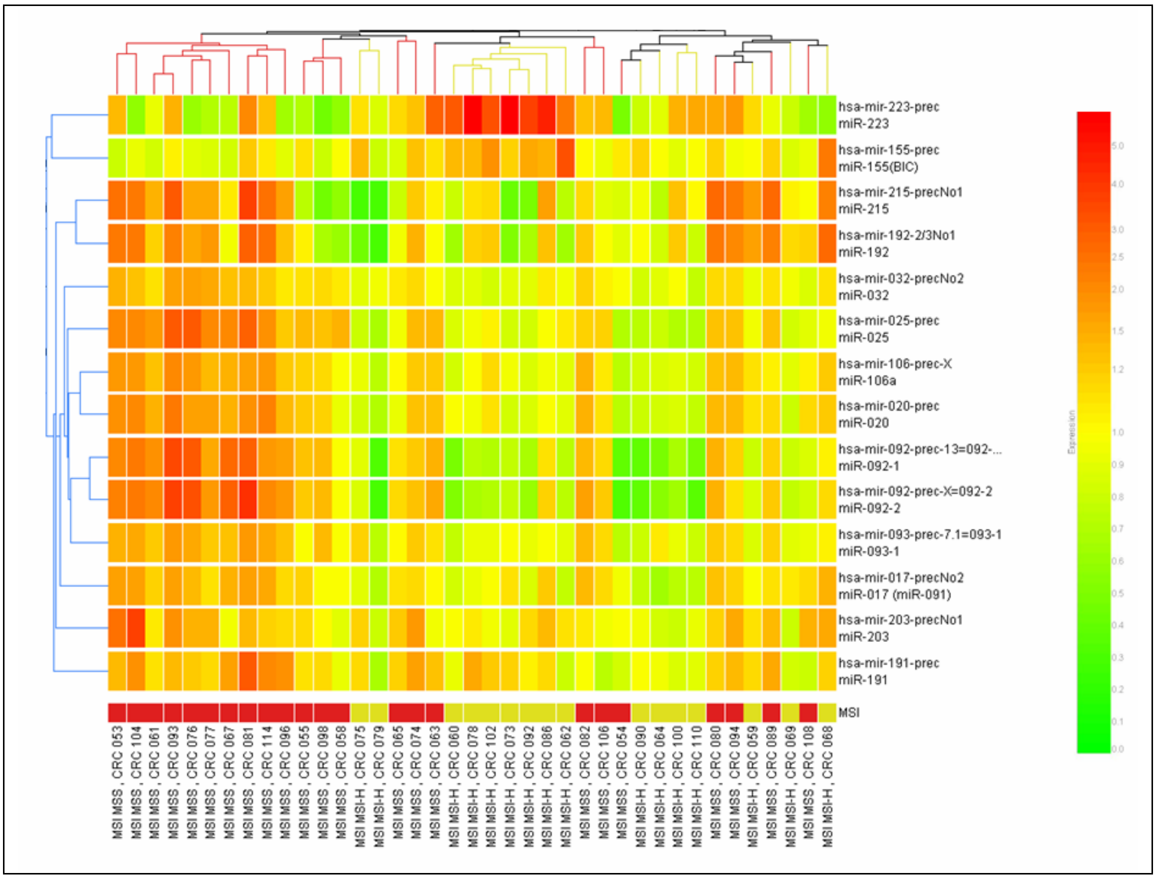

Additional file 4. Classification of tumors according to expression of microRNAs. Cluster of 39 colorectal tumors made with a list of 14 microRNAs differentially expressed between 23 microsatellite stable (MSS) CRCs and 16 CRCs with high microsatellite instability (MSI-H). About 75% of the sample is assigned to the correct group. MicroRNA red color means an expression value over the average across samples, green color the opposite.
