# Supplementary material for: mRNA/microRNA gene expression profile in microsatellite unstable colorectal cancer
Source: Mol Cancer. 2007 Aug 23;6:54. doi: 10.1186/1476-4598-6-54 (PMC2048978; doi:10.1186/1476-4598-6-54)
Supplement: Additional file 5 — Classification of tumors according to expression of 72 differentially expressed mRNAs. Cluster analysis based on differentially expressed mRNAs [file 1476-4598-6-54-S5.pdf]

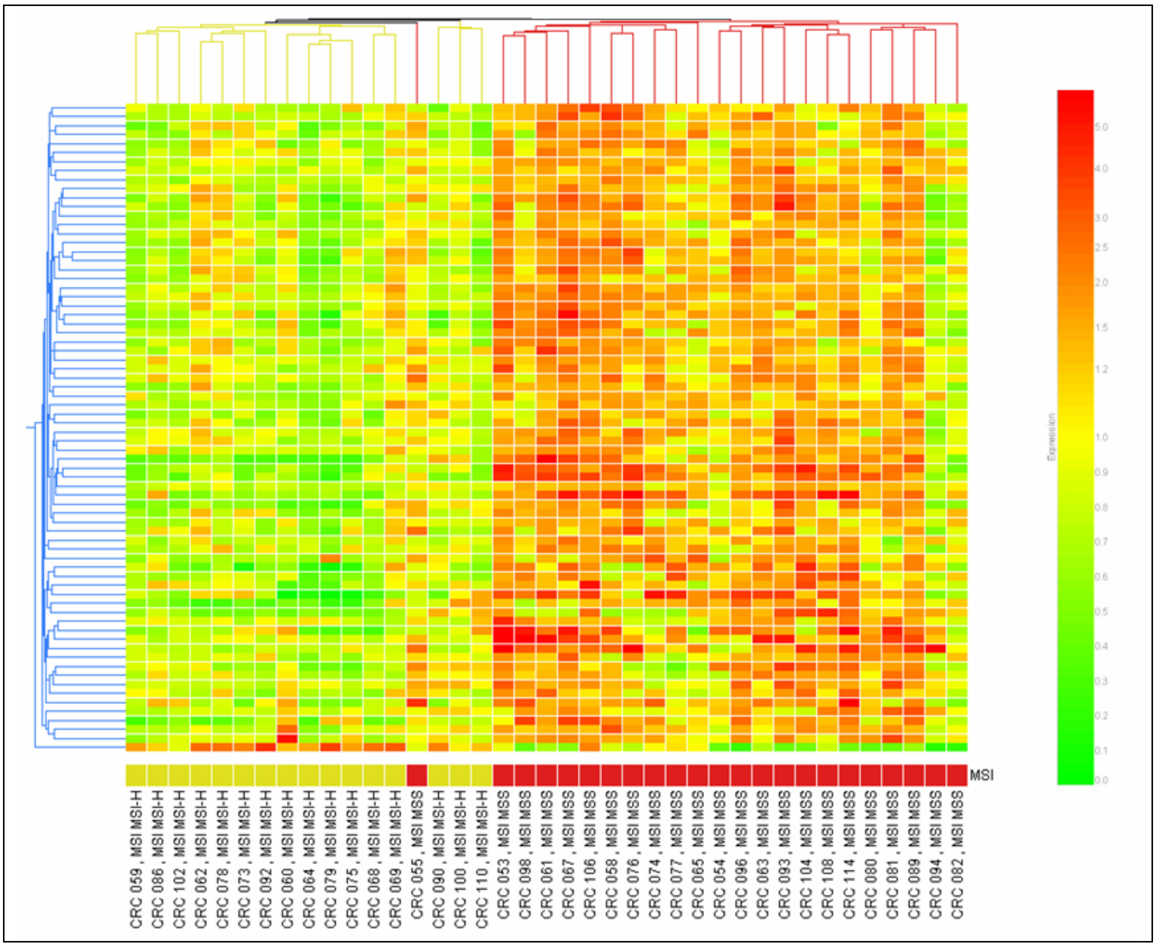

Additional file 5. Classification of tumors according to expression 72 differentially expressed mRNAs. Cluster of 39 colorectal tumors made with a list of 72 genes differentially expressed between 23 MSS CRCs and 16 MSI-H CRCs. One sample (CRC 55) is still wrongly classified. Genes red color means an expression value over the average across samples, green color the opposite.
