# Supplementary material for: mRNA/microRNA gene expression profile in microsatellite unstable colorectal cancer
Source: Mol Cancer. 2007 Aug 23;6:54. doi: 10.1186/1476-4598-6-54 (PMC2048978; doi:10.1186/1476-4598-6-54)
Supplement: Additional file 6 — Differentially expressed protein-coding genes shared with Di Pietro's study. List of protein-coding genes shared by this study and Di Pietro's study [file 1476-4598-6-54-S6.pdf]

**Additional file 6.** Differentially expressed protein-coding genes shared with Di Pietro's study

| Systematic | Gene Symbol | Description | MSI-H Normalized | MSS Normalized | p-value* |
|---|---|---|---|---|---|
| AL359056 | STX16 | syntaxin 16 | 1.08 | 1.33 | 4.91E-02 |
| NM_006253 | PRKAB1 | protein kinase, AMP-activated, beta 1 non-catalytic subunit | 3.09 | 4.87 | 4.56E-02 |
| NM_006144 | GZMA | granzyme A (granzyme 1, cytotoxic T-lymphocyte-associated serine esterase 3) | 2.75 | 1.46 | 4.56E-02 |
| NM_002740 | PRKCI | protein kinase C, iota | 1.14 | 1.74 | 4.02E-02 |
| AK026142 | GATAD1 | GATA zinc finger domain containing 1 | 1.12 | 1.59 | 3.84E-02 |
| X91648 | PURA | purine-rich element binding protein A | 2.07 | 3.00 | 3.81E-02 |
| NM_007127 | VIL1 | villin 1 | 9.87 | 29.14 | 3.00E-02 |
| NM_001445 | FABP6 | fatty acid binding protein 6, ileal (gastrotropin) | 0.92 | 1.39 | 2.26E-02 |
| NM_006420 | ARFGEF2 | ADP-ribosylation factor guanine nucleotide-exchange factor 2 (brefeldin A-inhibited) | 1.12 | 1.61 | 2.09E-02 |
| NM_005520 | HNRPH1 | heterogeneous nuclear ribonucleoprotein H1 (H) | 1.17 | 0.92 | 1.84E-02 |
| NM_007052 | NOX1 | NADPH oxidase 1 | 0.91 | 1.25 | 1.74E-02 |
| NM_002083 | GPX2 | glutathione peroxidase 2 (gastrointestinal) | 5.64 | 16.96 | 1.55E-02 |
| NM_004961 | GABRE | gamma-aminobutyric acid (GABA) A receptor, epsilon | 1.17 | 1.77 | 1.24E-02 |
| NM_004963 | GUCY2C | guanylate cyclase 2C (heat stable enterotoxin receptor) | 3.45 | 10.96 | 1.13E-02 |
| NM_005170 | ASCL2 | achaete-scute complex-like 2 (Drosophila) | 1.24 | 2.08 | 8.77E-03 |
| AK025683 | FARP1 | FERM, RhoGEF (ARHGEF) and pleckstrin domain protein 1 (chondrocyte-derived) | 1.42 | 2.26 | 2.81E-03 |
| L38517 | IHH | Indian hedgehog homolog (Drosophila) | 1.01 | 1.53 | 1.04E-03 |
| NM_012413 | QPCT | glutaminyl-peptide cyclotransferase (glutaminyl cyclase) | 0.82 | 1.41 | 9.35E-04 |
| NM_004485 | GNG4 | guanine nucleotide binding protein (G protein), gamma 4 | 0.94 | 1.68 | 6.47E-04 |
| NM_003878 | GGH | gamma-glutamyl hydrolase (conjugase, folylpolygammaglutamyl hydrolase) | 2.41 | 8.24 | 7.97E-05 |
| NM_002657 | PLAGL2 | pleiomorphic adenoma gene-like 2 | 1.01 | 1.81 | 2.91E-05 |

*P-value refers to the statistical comparison performed with our data set.
