# Supplementary material for: mRNA/microRNA gene expression profile in microsatellite unstable colorectal cancer
Source: Mol Cancer. 2007 Aug 23;6:54. doi: 10.1186/1476-4598-6-54 (PMC2048978; doi:10.1186/1476-4598-6-54)
Supplement: Additional file 7 — Gene Ontology classification based on Ingenuity Pathway Analysis. List of functional classes present in the differentially expressed protein-coding genes [file 1476-4598-6-54-S7.pdf]

Additional file 7. Gene Ontology classification based on Ingenuity Pathway Analysis

id	genes (a)	score	focus genes	top functions
1	ATR, BCL2, BLM, CCL5, CDX2, CFTR, CRX, CTNNB1, CYCS, DUT, E2F1, FANCF, GNLY, GPX2, GUCY2C, HMGB1, HSPE1, LBR, MLH1, MT1L, MUC6, PCCA, PTPN4, PURA, QPCT, SEMA5A, SFRS6, SQLE, SRPK1, STAU, SUV39H1, TDGF1, TOPBP1, WWOX, ZHX1	58	35	Cell Cycle, DNA Replication, Recombination, and Repair, Cell Morphology
2	ANKH, ARFGEF1, ARFGEF2, EXOSC5, EXOSC7, EXOSC8, FKBP2, FMR1, GNB1, GNG4, HSPE1, IGFBP6, IL15, IL20, IL20RB, IL22RA1, MKRN1, MLPH, MYO5A, MYO9B, MYRIP, POP1, POP7, PURA, RAB27B, RHOA, RPP14, RPP21, RPP30, RPP38, RPP40, RPSA, STAT3, TNF, VAV3	19	17	Gastrointestinal Disease, Genetic Disorder, Immunological Disease
3	ADRB1, CD86, FCGR1A, FCGR2B, FCGR3A, FEN1, FLJ20758, FOXC2, GZMA, HSPG2, HUS1, HUS1B, INPP5D, MASK, MBP, MPO, MT2A, NF2, PECAM1, PRKAR1B, PRSS23, RAB32, RAD1, RAD17, RAD9A, RAD9B, RECC5, RFC2, RFC3, RFC4, SERPINC1, SLC7A1, STK24, TAX1BP1, TNF	15	15	Inflammatory Disease, Skeletal and Muscular Disorders, Immune Response
4	AKT1, APEX1, ARG1, CALD1, CCND3, DKC1, DNTT, DUSP6, E2F5, G6PC, HNRPH1, HOXB4, HSPE1, IL6, KIAA0101, LAMP2, LEFTY2, MT1A, MYC, MYCBP, NOL5A, PCNA, POLD1, POLM, RAD18, RAMP1, RAX, RBP1, RUVBL2, TERC, TERT, TK1, UBE2C, XRCC5, ZIC2	15	15	Dermatological Diseases and Conditions, Genetic Disorder, DNA Replication, Recombination, and Repair
5	ADCYAP1, BTG2, CASK, CEBPD, CHUK, CSE1L, CTNNB1, DLGAP4, EPB41L1, FN1, FOS, GOSR1, GPC3, GPR30, GPSM2, GTF2IRD1, KIF23, MGAT3, MIA, NOL8, NSDHL, RCN1, RPSA, RRAGA, RRAGD, S100B, SFRP4, SNAP25, STK38, STX16, TBR1, TSC22D1, UBE2N, UBE2V1, VTI1B	15	15	Cancer, Cell Death, Cell Cycle

6	ARG1, BAK1, BIN1, CAMK4, CCR1, CD86, CDX2, CEBPA, DDIT3, DHFR, EGR2, EIF5B, FCER2, FCGR1A, GGH, GPR160, GRM8, GTF3A, GUSB, ID1, IFNG, IL12RB1, MYBL2, MYOD1, NAALADL2, NFYA, NFYB, NFYC, OAS2, SECTM1, SEPT4, SLC12A2, SMARCC1, TGOLN2, ZRF1	14	14	Gene Expression, Cellular Development, Hematological System Development and Function
7	ADK, AHCY, AREG, ASXL1, CLDN1, DOCK7, EED, EREG, F2, KIAA0853, KIAA1713, MARK3, MARK4, PARD3, PARD6A, PARD6B, PARD6G, PDCD6, PHF1, PHF19, PRKCI, PTPN3, RAF1, SYNPO2, TOMM7, TOMM20, TOMM22, TOMM34, TOMM40, TOMM70A, TPM1, TPM3, WNK2, YWHAB, YWHAZ	14	14	Cell-To-Cell Signaling and Interaction, Cellular Assembly and Organization, Protein Trafficking
8	ACSL5, APCDD1, CAMK2B, CDKN1B, COPS3, COPS6, COPS8, COPS7A, COPS7B, CSNK2A2, CYB5, DF, DVL2, FNTA, G6PC, GABRE, HNF4A, HPX, INS1, KHDRBS1, KHDRBS3, LEP, NEK6, PCSK1, PGGT1B, PRKAA1, PRKAA2, PRKAB1, PRKAG2, PRLR, SIRT1, SYT7, TLE1, WNT1, WNT11	14	14	Carbohydrate Metabolism, Small Molecule Biochemistry, Lipid Metabolism
9	ADAMDEC1, ATF3, BAG1, C5, C6ORF79, CASP3, CD72, CHN2, DCC, GAS6, GAS7, GPX4, HMGB1, HSPB1, HSPBAP1, IL8, IL13, IL1R1, IL8RB, LOC93081, MPO, PI3, PRKRIR, PRTN3, SIAH1, SLC25A15, SLPI, SNCA, SNCAIP, SPHK1, ST13, STK4, SYP, TFF3, WNT5A	12	13	Cell-To-Cell Signaling and Interaction, Hematological System Development and Function, Immune and Lymphatic System Development and Function
10	ARG1, ASCL2, BNIP3L, CCND1, CCNE2, CCR3, CREBBP, CXCL3, DACH1, EGR1, EYA3, FGF5, GMEB1, GMEB2, IER2, IHH, IL3, KIAA1008, KLF10, KLF13, NR3C2, NUP153, NUP214, NXT1, PCSK7, PROX1, RAN, RBL2, SUV39H1, TGFB1, TRAF5, UBTF, USF2, XPO5, ZNF183	12	13	Cellular Development, Skeletal and Muscular System Development and Function, Gene Expression
11	ACTL6A, C20ORF20, EP400, GAS2, GEMIN5, GEMIN6, GEMIN7, HRAS, IGF2, MCM2, MCM4, MORF4L1, NDRG1, PIAS4, PLAGL2, POLA, PRIM1, PRIM2A, RAD54B, RPL39, RPS6KA3, SERPINB2, SHFM1, SIP1, SIVA, SMARCB1, SMARCE1, SMN1, TAF1A, TAF1B, TAF1C, TP53, TRIM44, TRRAP, YEATS4	11	12	RNA Post-Transcriptional Modification, Cellular Assembly and Organization, Viral Function

12	ARNTL, BIN1, CBX3, CBX5, CLOCK, CP, CSNK1E, DBP, EIF4E, ELF3, EN2, FABP6, FCER2, HAMP2, IL1B, NFATC2, NGFB, NPAS2, NR0B2, NR1H4, P2RX7, PLD1, PTPRK, RRS1, RXRA, SERPINF1, SLC3A1, SLC3A2, SLC40A1, TAF4, TAF7, TAF11, TGFBR2, TGFBRAP1, TIF1	10	11	Gene Expression, Behavior, Nervous System Development and Function
13	ADM, CACNA1A, CACNA1B, CACNA1C, CACNA1D, CTSD, FGF4, FGFR3, FOXO3A, GAP43, GRN, GTF2I, IGF1, IGFBP1, IL11, KCNMA1, KRT5, MAPK1, NOX1, NPHP1, NRG4, NRGN, PLCG1, PLCG2, PTK2B, PTPRJ, PTPRO, SGK, SGK2, SGKL, SH2B, SIRT1, SYT2, VEGF, VIL1	8	10	Post-Translational Modification, Cellular Growth and Proliferation, Cell Morphology
14	ATR, ATXN3, BARD1, BRCA1, CBFA2T2, CBFA2T3, CDK8, CPSF3, CRSP4, CRSP5, CRSP7, CRSP9, CSTF2, CTDP1, CXORF53, HDAC6, HEAB, HNRPFL, MED4, MED12, MED25, MED28, MED31, NCBP1, NCBP2, NSFL1C, PCQAP, RUNX1T1, SURB7, SURF5, THRAP1, THRAP5, TSEN2, VCP, VCPIP1	7	9	Gene Expression, Cell Signaling, RNA Post-Transcriptional Modification
15	ESRRBL1, IFT20, IFT52, IFT88	3	2	Cellular Assembly and Organization, Cell Morphology, Cellular Function and Maintenance
16	C20ORF172, ZWINT (HZwint-1)	1	1	Cell Cycle, Cellular Assembly and Organization, DNA Replication, Recombination, and Repair
17	BUB1, SGOL1	1	1	Cellular Assembly and Organization, Cell Cycle, DNA Replication, Recombination, and Repair
18	BLCAP, TFAP2A	1	1	Gene Expression, Organismal Development, Cancer
19	TFIP11, TUFT1	1	1	
20	MDM2, MTBP	1	1	Cancer, Cell Cycle, Skeletal and Muscular Disorders

21	IRF5, ZNF443	1	1	Cancer, Cell Death, Gastrointestinal Disease
22	PEX16, PEX19	1	1	Genetic Disorder, Metabolic Disease, Cellular Assembly and Organization
23	FTH1, VMD2	1	1	Amino Acid Metabolism, Drug Metabolism, Genetic Disorder
24	MGL, SN	1	1	Immune Response
25	HPS1, HPS4	1	1	Genetic Disorder, Cellular Development, Hair and Skin Development and Function
26	NDFIP2, NEDD4	1	1	Post-Translational Modification
27	NUDCD3, TCOF1	1	1	Developmental Disorder, Genetic Disorder, Gene Expression
28	PRKCBP1, ZFYVE9	1	1	Post-Translational Modification, Cell Morphology, Cellular Assembly and Organization
29	CETN2, RAD23B, XPC	1	1	DNA Replication, Recombination, and Repair, Nucleic Acid Metabolism, Small Molecule Biochemistry
30	LNX, LNX2, NUMB, NUMBL	1	1	Protein Synthesis, Cellular Growth and Proliferation, Embryonic Development

(a) 212 of 451 genes are annotated
